# High-throughput sequencing of small RNAs revealed the diversified cold-responsive pathways during cold stress in the wild banana (*Musa itinerans*)

**DOI:** 10.1186/s12870-018-1483-2

**Published:** 2018-11-29

**Authors:** Weihua Liu, Chunzhen Cheng, Fanglan Chen, Shanshan Ni, Yuling Lin, Zhongxiong Lai

**Affiliations:** 1 Institute of Horticultural Biotechnology, Fujian Agriculture and Forestry University, Fuzhou, 350002 China; 2 Chongqing Normal University, Daxuecheng Middle Rd, Chongqing, Shapingba Qu China

**Keywords:** *Musa itinerans*, Cold stress, RNA-seq-based profiling, miRNA, miR172

## Abstract

**Background:**

Cold stress is one of the most severe abiotic stresses affecting the banana production. Although some miRNAs have been identified, little is known about the role of miRNAs in response to cold stress in banana, and up to date, there is no report about the role of miRNAs in the response to cold stress in the plants of the cultivated or wild bananas.

**Result:**

Here, a cold-resistant line wild banana (*Musa itinerans*) from China was used to profile the cold-responsive miRNAs by RNA-seq during cold stress. Totally, 265 known mature miRNAs and 41 novel miRNAs were obtained. Cluster analysis of differentially expressed (DE) miRNAs indicated that some miRNAs were specific for chilling or 0 °C treated responses, and most of them were reported to be cold-responsive; however, some were seldom reported to be cold-responsive in response to cold stress, e.g., miR395, miR408, miR172, suggesting that they maybe play key roles in response to cold stress. The GO and KEGG pathway enrichment analysis of DE miRNAs targets indicated that there existed diversified cold-responsive pathways, and miR172 was found likely to play a central coordinating role in response to cold stress, especially in the regulation of *CK2* and the circadian rhythm. Finally, qPCR assays indicated the related targets were negatively regulated by the tested DE miRNAs during cold stress in the wild banana.

**Conclusions:**

In this study, the profiling of miRNAs by RNA-seq in response to cold stress in the plants of the wild banana (*Musa itinerans*) was reported for the first time. The results showed that there existed diversified cold-responsive pathways, which provided insight into the roles of miRNAs during cold stress, and would be helpful for alleviating cold stress and cold-resistant breeding in bananas.

**Electronic supplementary material:**

The online version of this article (10.1186/s12870-018-1483-2) contains supplementary material, which is available to authorized users.

## Background

Banana belongs to the genus *Musa*, a member of the family *Musaceae*, is divided into two subgroups, i.e., the sweet or dessert banana, and the cooking banana, both of which are the most popular fruit in the world, and they are also vital for food security in many tropical and subtropical countries [1–3]. With some 15% of global production exported, its total trade value stood at some USD 8 billion in 2016, making bananas the largest traded fruit crop in value terms [4]. China is one of the major production regions of banana in the world, and the production amount of banana approximately accounts for 10% of the global banana production amount per year.

Banana plants are thought to be thermophilic crops that distribute in the warm tropical or subtropical regions. In general, they grow well in a temperature range of 15 °C–35 °C, and the cultivated banana plants generally stop growing when the temperature drops below 10~ 17 °C depending on the cultivars/varieties [5–7], and for most banana cultivars in China, the critical temperature is thought to be 13 °Cor so [8]. In China, most of banana productions are in the southern subtropical regions that are at the northern margin of world banana cultivation. The cultivated banana plants here thus often suffer chilling or freezing injuries (in chilling, water-freezing temperature even plant-freezing temperature) in winter and even in spring when extreme weather events occur. Banana researchers and growers have been trying to explore banana cold-resistant genes and developing cold-resistant banana cultivars or to protect the banana from chilling or freezing by mean of cold-proof measures [9]; to date, however, effective methods to solve chilling or freezing injury problems of banana have not yet been satisfactory [10].

As a result of evolution, there exist traits for cold, chilling and freezing tolerances in the wild genetic resources that are of potential value for crop breeding [2, 11]. The wild banana genetic resources are abundant in China, and some of them are cold tolerant according to the studies over the past decades [12, 13]. Based on screening the wild banana genetic resources collected by our lab, a wild banana line, ‘Sanming wild banana’ (*Musa itinerans* ‘Sanmingyeshengjiao’), was found at Sanming City, Fujian Province of China. It is extremely cold tolerant as it grows well around 0 °C [12], and its semilethal temperature reached as low as − 1.776 °C [14]. Thus, it is of great usefulness for banana cold-resistance breeding [10, 14], and is consequently used as material in the present studies.

MicroRNAs (miRNAs), are posttranscriptional regulators of gene expression [15–17], which regulate biological processes, such as development, growth and stress responses [18–21]. In plants, the transcriptional control of the expression of cold-responsive genes is well known [22], but miRNAs have recently been added to the suite of cold-responsive gene regulatory networks [19], which play a significant role in cold stress in plants [20, 21]. Since 2010, high-throughout sequencing has been applied to the discovery of miRNAs in more and more species of plants, and the miRNA profiles in response to plant cold stress were revealed, which included *Arabidopsis* [23], tomato [24–26], celery [27], *Triticum aestivum* [28], cassava [29, 30], soybean [31], *Camellia sinensis* [32, 33], almond [34], grapevine [35], litchi [36], *Citrullus lanatus* [37], *Medicago sativa* [38], etc. Moreover, remarkable progress have been made in the applications of high-throughout sequencing to profiling miRNAs in response to other kinds of plant stresses, especially in the discovery of miRNAs related to drought/dehydration stress in crops, such as cereals [39–41], including wheat [42–44], *Triticum dicoccoides* [45], barley [46], and grasses, including *Festuca arundinacea* [47] and *Brachypodium distachyon* [48], etc. More more reports suggested that RNA-seq should provide a new insight in the stress-responsive miRNAs in plants, since stress (including cold and drought stresses) responsive miRNAs are specific to some extent in different species, and exploring the stress responsive miRNAs in each plant species is necessary. Up to date, the function of the stress responsive miRNAs in bananas remains unknown.

In the present research, the Illumina solexa sequencing, qPCR validation and some physiological analyses were conducted in the wild banana (*Musa itinerans* ‘Sanmingyeshengjiao’) plants grown in different low temperatures.

## Results

### Determination of cold tolerance of the wild banana

**Fig. 1:**
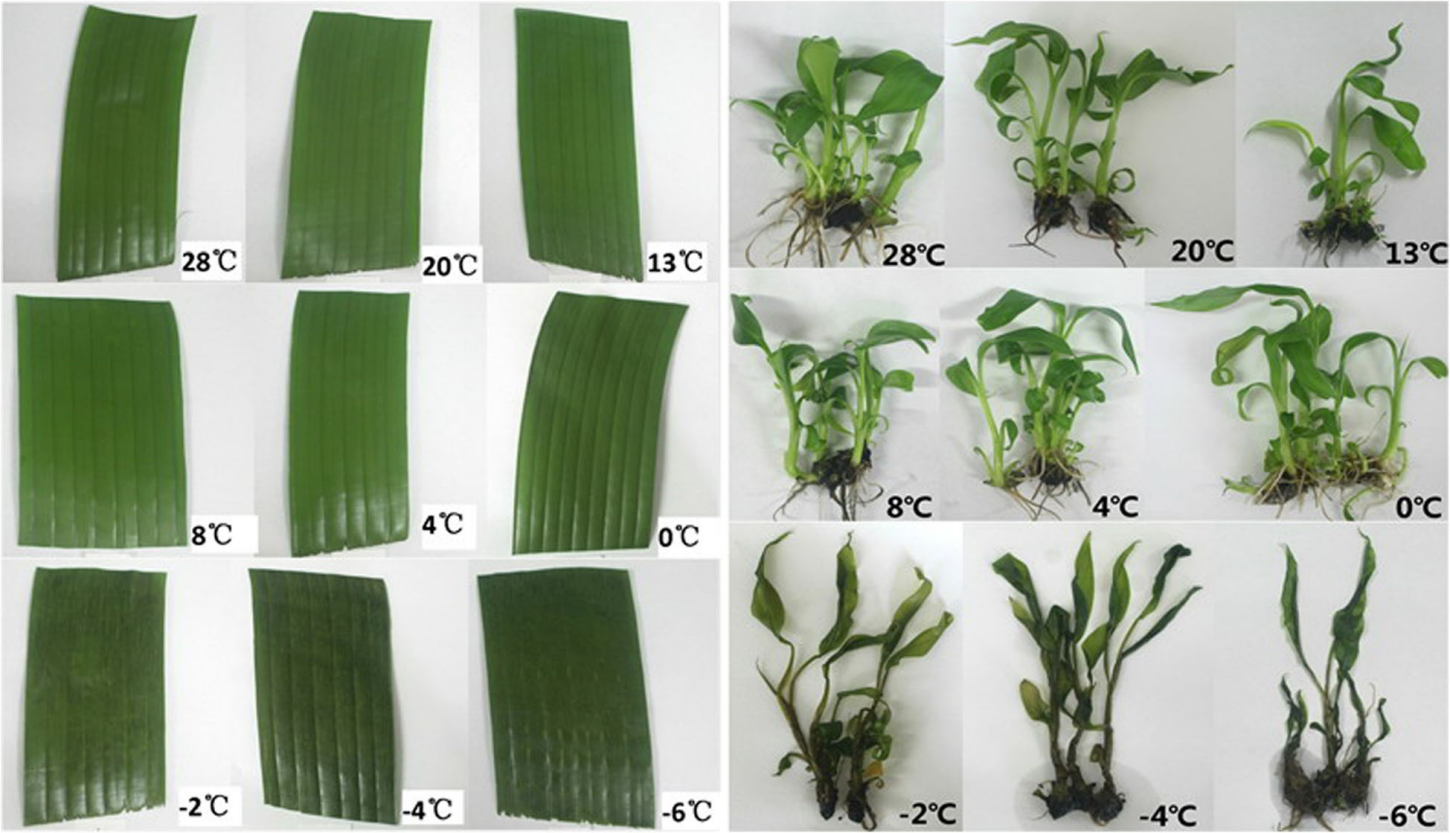
Morphological changes of leaves from field plants and in vitro plants treated during cold stress

The wild banana *Musa itinerans* ‘Sanmingyeshengjiao’ has been discovered by our lab for over 10 years, and it grows naturally in the region of Sanming City, where the extreme low temperature even reached - 4 °C in 2016 (Additional file 1) according to the data provided by the local Meteorological Agency of Sanming city for the recent 5 years. Recently, our lab reported that the semilethal temperature of the wild banana reached − 1.776 °C, which suggested that it should be lower than those of the common bananas and cold-resistant [14]. For further verification of the cold tolerance of the wild banana, the leaves from the field plants and the in vitro cultured plants were placed at various temperature conditions, and the results showed that the morphological changes of the leaves from both the field plants and the in vitro plants were similar in all the temperature conditions tested (Fig. 1). The in vitro plantlets or leaves appeared normally under the treatment of 0 °Cand beyond 0 °C; however, the in vitro plantlets or leaves showed heavy damages, such as serious water-logging, even wilting or death under the treatment of - 2 °C and below - 2 °C. Therefore, the wild banana was cold-resistant, and it was further used for exploring profiling of the cold-responsive miRNAs by RNA-seq in this experiment.

### Distribution of small RNAs categories and their sizes in *Musa itinerans*

To identify miRNAs involved in the process of cold stress of the plants in the wild banana (*Musa itinerans* ‘Sanmingyeshengjiao’), the 4 small RNA (sRNA) libraries were generated from the 4 samples of leaves after different low-temperature treatments. The SRA accession number of the raw data is SRP148506. After removing the low quality (N% > 10%, or with ploy A/T/G/C) and adapter sequences from raw data obtained by sequencing, the numbers of the clean reads of the 4 samples were 13,021,737(0 °C), 13,021,737 (4 °C), 12,985,175 (13 °C) and 11,056,016 (28 °C), respectively (Additional file 2).

The sequenced small RNAs from the 4 libraries included known miRNAs (0.39%~ 4.43%), novel miRNAs (0.003%~ 0.10%), small nuclear RNA (snRNA), small nucleolar RNA (snoRNA), rRNA, tRNA, repeat, NAT, TAS (ta-siRNA), exon:+, exon:-, intron:+ and other (40.39%~ 46.84%, unannotated). The known miRNAs (reads) of 147,854 (0 °C), 180,184 (4 °C), 146,370 (13 °C), 12,996 (28 °C) and the novel miRNAs of 1526 (0 °C), 3945 (4 °C), 1349 (13 °C), 106 (28 °C) were predicted when the Banana Genome A was used as the reference (Additional file 3: Table S2–S1). Surprisingly, the read percents of known miRNAs, novel miRNAs and TASs were very small (0.39%, 0.00315% and 0.00054%) in the control of 28 °C, but in the cold treated groups, they were as high as 4.10%~ 4.43% (10 times of the control), 0.04%~ 0.10% (15–37 times of the control) and 0.01% (18–25 times of the control), respectively, and all of them reached the peak at 4 °C, which suggested that the miRNAs and TASs, particularly the novel miRNAs and TASs, play essential roles in response to the cold stress, especially in chilling.

**Fig. 2:**
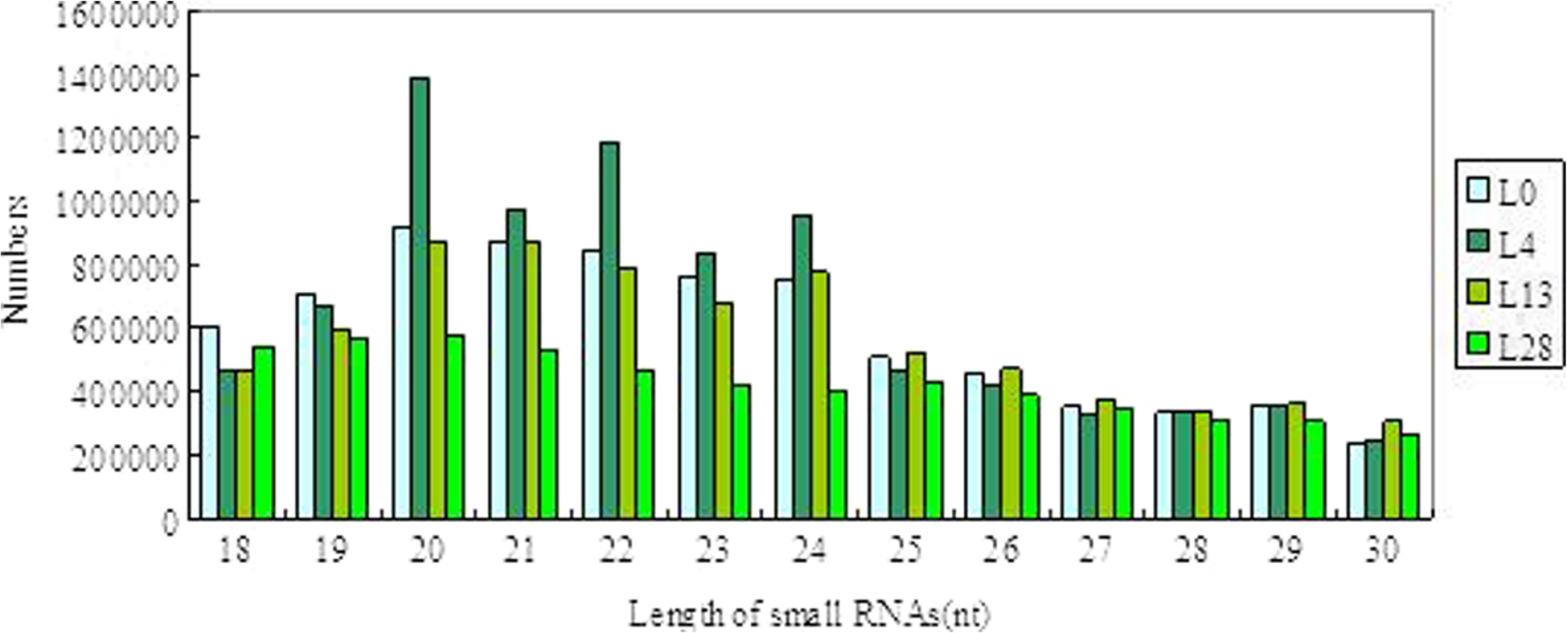
The length distribution of sequencing reads from 4 small RNA libraries in the wild banana. L0, L4, L13, L28: treated at 0 °C, 4 °C, 13 °C, 28 °C, respectively (the same below)

In general, sRNAs range from 18 to 30 nucleotides (nt) in size, and the majority of mature miRNAs are 21 nt in plants. The distribution of small RNA categories and their sizes in *Musa itinerans* were shown in Fig. 2 and Additional file 4. The numbers of different sizes of sRNAs differed from different temperature treatments, and the majority of them were 20 nt in size, followed by 21 nt, 22 nt, 23 nt, 24 nt. However, Bi et al. (2015) [49] reported the majority of sRNAs were 23 nt in size during fruit ripening, and they indicated that the differences resulted from tissue-specific expression under different treatments; Wen et al. (2014) [50] reported the majority of sRNAs were 21 nt in size, followed by 24 nt, 22 nt and 20 nt in the leaf of banana. In the former reports, the length of plant sRNAs varied from 20 to 24 nt, such as the majority of sRNAs were 24 nt in *Arabidopsis* [51], *Medicago truncatula* [52], maize [53], potato [54], tomato [55], *Dimocarpus longan* [56]; 23 nt in *Cucumis sativus* [57]; 22 nt in sugarcane [58]; 21 nt in wheat, Chinese yew and grapevine [59–61]. Interestingly, in this experiment, the distributions of sRNAs were the similar in size between the treatment of 0 °C and 28 °C, i.e., the majority of sRNAs were 20 nt, followed by 21 nt, 22 nt, 23 nt, 24 nt. Therefore, we suggested that, the distributions of sRNAs in size vary in the different species/cultivars and tissues, or under abotic/biological stress, as well as by other factors.

### Known miRNAs, novel miRNAs and trans-acting siRNA (ta-siRNAs) identified in the wild banana during low temperature stress

Mapped to the Banana Genome A against miRBase22, the results showed as additional files for the known miRNAs (Additional file 5), novel miRNAs (Additional file 6) and ta-siRNAs (Additional file 7). The numbers of mature miRNAs and hairpins were respectively 265 and 596 mapped to the Banana Genome A without mismatch. The unique sRNAs (2844) and total sRNAs (487,404) were observed in different temperature treatment groups (Additional file 3 Table S2–2). The highest levels of the numbers of the mature miRNAs, hairpins, unique sRNAs and total sRNAs occurred at 4 °C, and the lowest were at 28 °C.

Further analysis revealed that the 265 known miRNAs, belonging to 43 miRNA families, varied from 18 nt to 25 nt in size mapped to the Banana Genome A (Additional file 5). 28 miRNA families contained more than one members, including the families of miR396, − 171, − 156, − 166, − 159, − 172, − 169,-167, − 395,-397, − 160, − 319, − 164, − 408, − 398, 399, − 390, − 393, − 162, 530, − 535, − 157, − 3630, − 168, − 1511, − 444, − 845, − 858. 15 miRNA families contained one member, including miR394, miR3711, miR4995, miR5083, miR5179, miR528, miR529, miR5139, miR5368, miR5532, miR5538, miR6300, miR6478, miR8155, miR827, respectively. There were 41 novel miRNAs mapped to the Banana Genome A, and interestingly, among the novel miRNAs, some miRNA* (star miRNA) existed as many as 4–16 in number (Additional file 3), implying that both strands of these duplexes might be equally incorporated into the RISC [62]. The numbers of miRNA* were also the lowest at 28 °C, and they showed higher levels remarkably at all the cold stress temperatures, which suggested that the miRNA* also play roles in response to cold stress in the wild banana.

Among the novel miRNAs, the total numbers of mature miRNAs, star miRNAs and hairpins were 41, 16, 41, respectively. Meanwhile, the total numbers of novel unique sRNAs and total novel sRNAs of 475 and 6926 were mapped (Additional file 3 Table S2–3). Moreover, the numbers of the novel mature miRNAs, hairpins, unique sRNA and total sRNA were also the highest at 4 °C, and the lowest at 28 °C for the control, being similar to the numbers of sRNA reads as well as the numbers of the known miRNAs.

Ta-siRNAs were a class of small interfering RNA (siRNA) that repress gene expression by post-transcriptional gene silencing in plants, which were originally detected in 2004 in *Arabidopsis thaliana* [63]. In this experiment, mapped to the Banana Genome A against TAS.rc, the ta-siRNAs were predicted by UEA sRNA tools [64] and the results were showed as Additional file 7. The species of TASs peaked at 4 °C, the numbers of TASs were the highest at 13 °C, and the species and numbers of TASs were both the lowest at 28 °C, which was almost in accordance with TAS read numbers.

### Analysis for the DE miRNAs

Comparisons of the expression levels of miRNAs from RNA-seq data were carried out by DEG seq package [65] to identify DE miRNAs during cold stress in the wild banana. In the prediction of the numbers of DE miRNAs, qvalue< 0.005&|log2 (foldchange)| > 1 was set as the threshold for controlling the false positives.

**Fig. 3:**
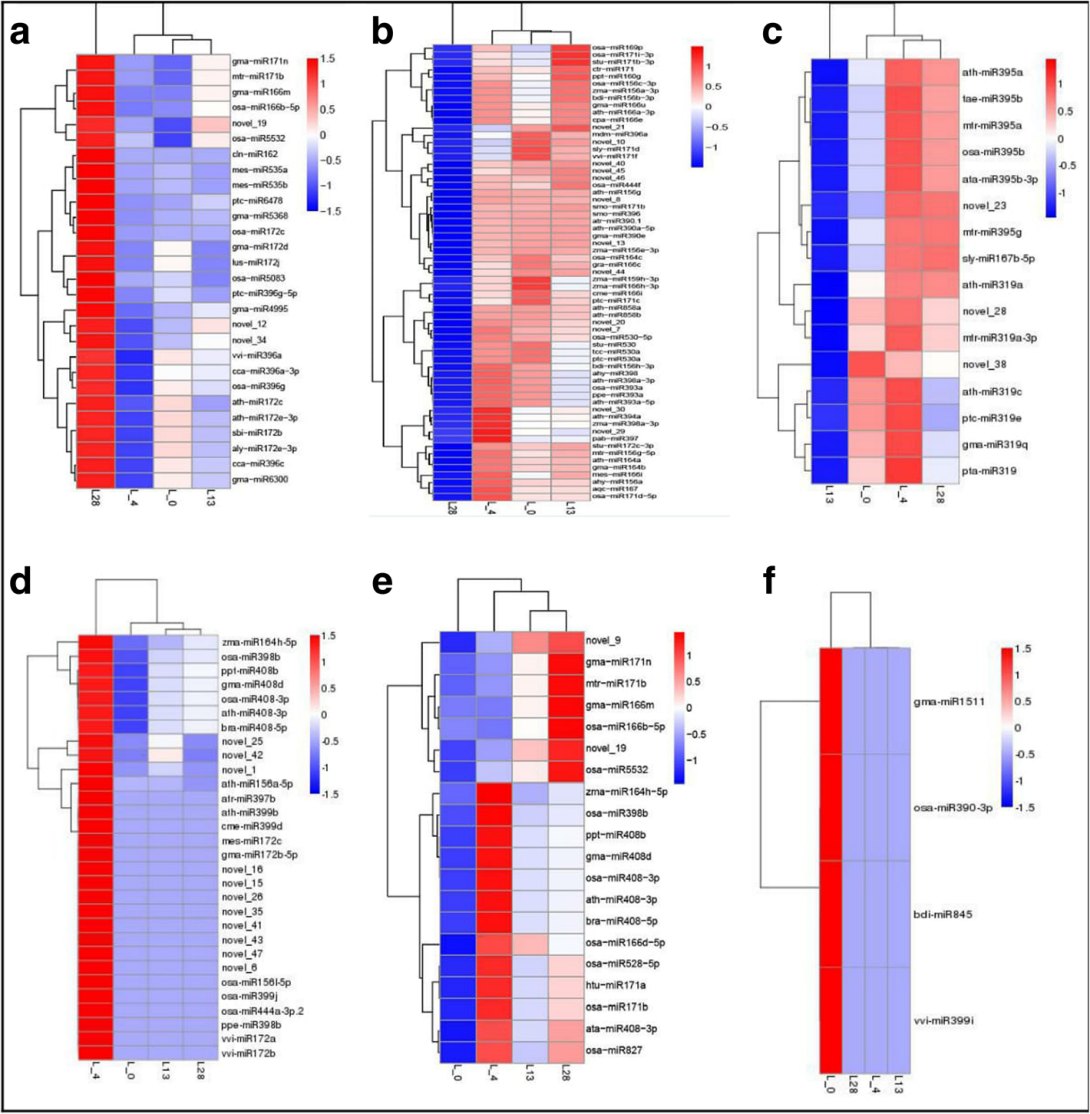
Heatmap of DE miRNAs during cold stress in the wild banana. The color represents miRNA expression values from 1.5 (the red corresponds to sRNAs with high expression) to − 1.5 (the blue corresponds to sRNAs with low expression). L0, L4, L13 and L28 correspond to the libraries obtained in the temperature 0 °C, 4 °C, 13 °C and 28 °C respectively. **a** up-regulation at 28 °C but down-regulation at other 3 temperatures; **b**: down-regulation at 28 °C but up-regulation at other 3 temperatures; **c**: down-regulation at 13 °C but up-regulation at other 3 temperatures; **d**: up-regulation at 4 °C but down-regulation at other 3 temperatures; **e**: down-regulation at 0 °C but up-regulation at other 3 temperatures; **f**: up-regulation at 0 °C but down-regulation at other 3 temperatures

Cluster analysis of DE miRNAs was conducted and the results showed in the Additional file 8 (the overview) and Fig. 3.

According to the cluster result, all the DE miRNAs were assigned to 3 main clades, which showed 3 changing patterns of predicted DE miRNAs under cold stress. In the first clade, the miRNAs showed down-regulated at the normal growth temperature of 28 °C, and up-regulated during cold stress (13 °C, 4 °C and 0 °C). In the second clade, the miRNAs showed up-regulated at the normal growth temperature of 28 °C, but most of them showed down-regulated during cold stress (13 °C, 4 °C and 0 °C). In the third clade, the miRNAs showed up-regulated at the chilling temperature (4 °C), and down-regulated at the other temperatures (28 °C, 13 °C, 0 °C), which suggested that these miRNAs be involved in chilling response in the wild banana.

**Fig. 4:**
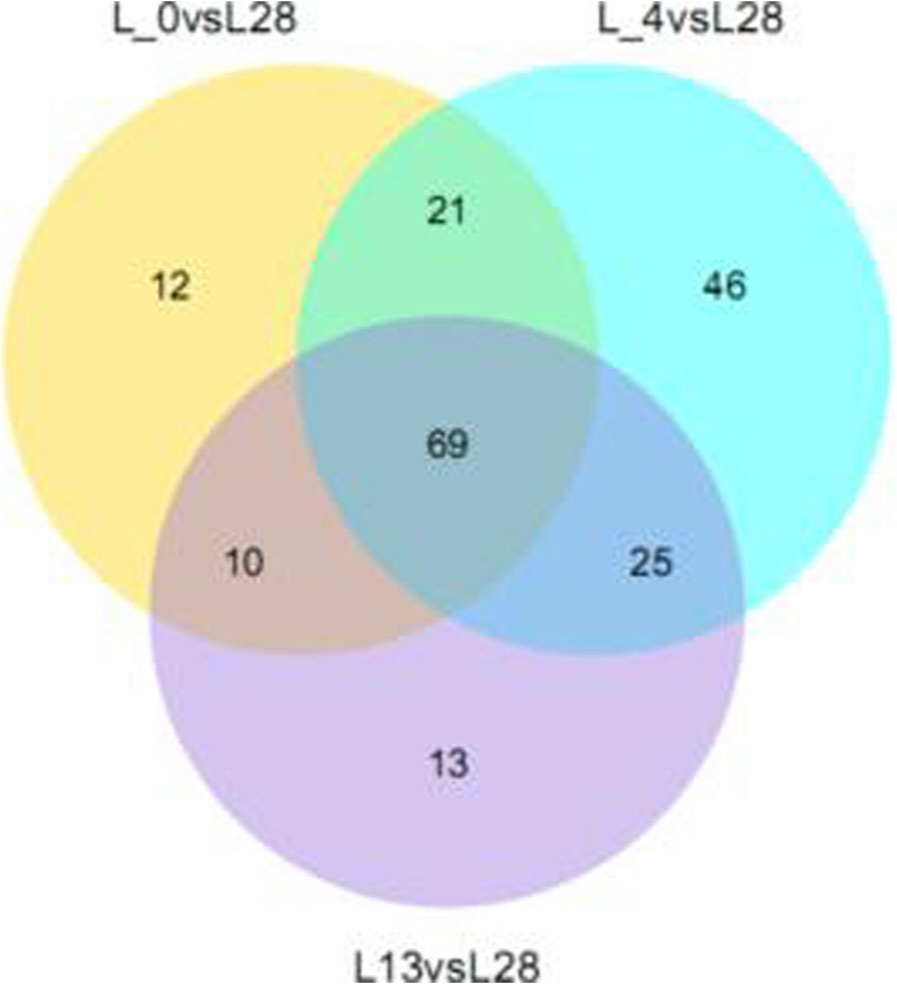
Venn diagrams of DE miRNAs among 3 groups during cold stress in the wild banana

The venn diagrams of the DE miRNAs between L4 vs L28 and L0 vs L28, L13 vs L28 and L0 vs L28, L4 vs L13 and L0 vs L13, L4 vs L13 and L4 vs L0, L4 vs L28 and L13 vs L28, L0 vs L28 and L4 vs L28 were showed as Additional file 9, and the venn diagram of the DE miRNAs among L0 vs L28, L4 vs L28 and L13 vs L28 was showed as Fig. 4. The data showed that the 69 DE miRNAs were presented at all the groups, which suggested that the 69 miRNAs should be involved in chilling and 0 °C treated responses, and 12, 46 and 13 miRNAs should be specifically in response to 0 °C, 4 °C and 13 °C, respectively, in the wild banana.

There were 41 novel miRNAs mapped to the Banana Genome A. TPM analysis indicated that at 4 °C, 23 novel miRNAs showed the highest expression levels accounting for 56%, and 9 of them expressed only at the temperature. At 0 °C, 4 novel miRNAs showed the highest expression levels accounting for 56%, and one of them expressed only at this temperature. At 13 °C, 7 novel miRNAs showed the highest expression levels, which was the same at 28 °C. Therefore, the highest expression levels occurred in 34 novel miRNAs, accounting for 83%, at all the cold temperatures (0 °C, 4 °C, 13 °C), and 26 of the 41 novel miRNAs did not express at 28 °C, which were very worth to further explored.

### Prediction and functional analysis of the targets for the DE miRNAs

To better understand the biological functions of the wild banana miRNAs in response to cold stress, the putative targets of the known and novel mature miRNAs were respectively predicted in the wild banana. The predicted putative target gene numbers were 2379 against the Banana Genome A (Additional file 10). According to the target prediction, it was found that in the conserved miRNAs, abiotic stresses were related, e.g., sulphate adenylyltransferase for miR395, uperoxide dismutase, copper/zinc binding domain for miR398, laccase for miR397 and miR408. On the other side, novel miRNAs were found where transcription factors (TFs) were related, e.g., quamosa promoter binding protein box (SBP-box) for novel_23, cytochrome P450 for novel_8, Na^+^/H^+^ exchanger for novel_2. Additionally, from the point of view of function, one miRNA could regulate one or multiple targets. In the meanwhile, a few miRNAs might regulate only one target, such as ptc-miR6478 targeting galactose oxidase, novel_30 targeting RNA helicase, ATP-dependent, SK12/DOB. Most of the miRNAs were found to regulate more than 10 targets, and some miRNAs to regulate at least 100 targets, especially, miR156 family had 2362 targets, miR172 family had 1060 targets, miR396 family had 1023 targets, miR171 family had 454 targets, miR169 family had 271 targets, miR397 family had 256 targets. The above findings suggested that miRNAs were widely involved in biological processes during cold stress in the wild banana. Nevertheless, a single target could be regulated by multiple miRNAs, e.g., glycosyl transferase and myb-domain genes were likely to be regulated by as many as 25 miRNAs each. Thus, the results implied that the miRNA regulation was complicated and intervolved forming a cold response complex network during cold stress in the wild banana (Additional files 10, 11).

Moreover, GO and KEGG pathway enrichment analysis was conducted to functionally categorize the target genes. The targets of the DE miRNAs for each group (L0 vs L28, L0 vs L13, L13 vs L28, L4 vs L13, L4 vs L28, L4 vs L_0) were respectively categorized by GO and KEGG pathway enrichment analysis.

The total numbers of terms, which were enriched under the three main GO categories, were mapped to the Banana Genome A under qvalue< 0.05 (Additional files 12, 13). The numbers of terms enriched in BP category were the most in the groups of L4 vs L0, L4 vs L28, L13 vs L28, and the numbers of terms enriched in MF category were the most in the groups of L4 vs L13, L0 vs L13; however, the number of terms enriched in BP category was the most in L0 vs L28. The numbers of terms enriched in CC category were the least in the six groups. In L0 vs L13, there were no targets enriched in CC category. The GO enrichment of L4 vs L13, L0 vs L13 and L0 vs L28 exhibited significant differences (Additional file 12-d, e, f).

All the putative target genes for DE miRNAs were enriched to the KEGG pathway (Additional file 14), and the top 20 enriched pathways were showed as Table 1 and the details were showed in Additional file 15. Interestingly, some pathways occurred only in one group, for example, circadian rhythm-plant pathway occurred only in L0 vs L28, implying that the circadian rhythm maybe played specific roles in response to 0 °C treated temperature; the photosynthesis pathway, glyoxylate and dicarboxylate metabolism, fructose and mannose metabolism, phosphatidylinositol signaling system, lysine biosynthesis pathways occurred only in L0 vs L13, implying that the 5 pathways were essential from the critical growth to the 0 °C treated temperature in the wild banana, and so many specific pathways occurred suggested that the biological process remarkably change between them; the galactose metabolism and fatty acid degradation pathways occurred only in L4 vs L0, implying the 2.

**Table 1:** The top 20 KEGG pathways enriched by target genes of DE miRNA in 6 groups

pathway_term	group	pathway_term	group	pathway_term	group
Sulfur metabolism	★◆▲●▼■	Purine metabolism	★◆▲●▼■	Selenocompound metabolism	★◆▲● ■
Citrate cycle	★◆▲●▼	Sulfur relay system	★◆ ●▼	Peroxisome	★ ▲ ▼■
Endocytosis	★◆ ▼■	ABC transporters	★◆ ●▼	mRNA surveillance pathway	★ ▲ ▼■
Cysteine and methionine metabolism	★◆ ●▼	Nicotinate and nicotinamide metabolism	★◆▲ ■	Glycerophospholipid metabolism	★◆ ●
RNA polymerase	▲ ▼■	Ether lipid metabolism	★◆ ■	Sphingolipid metabolism	★◆ ●
Pyruvate metabolism	▲ ▼■	Carbon metabolism	◆▲●	Pyrimidine metabolism	▲● ■
Propanoate metabolism	▲ ▼■	Other glycan degradation	★ ▲ ■	Aminoacyl-tRNA biosynthesis	★◆ ●
Synthesis and degradation of ketone bodies	▲ ▼■	Sesquiterpenoid andtriterpenoid biosynthesis	◆▲ ■	Ribosome biogenesis in eukaryotes	▲ ▼■
Spliceosome	◆ ●	Oxidative phosphorylation	★ ●	Carotenoid biosynthesis	▲ ▼
RNA degradation	▼■	Biosynthesis of amino acids	★◆	Glycosaminoglycan degradation	▼■
SNARE interactions in vesicular transport	◆ ●	Protein export	▼ ■	Fructose and mannose metabolism	●
Fatty acid degradation	★	Plant-pathogen interaction	◆	Lysine biosynthesis	●
Butanoate metabolism	▲	Fatty acid biosynthesis	▲	Steroid biosynthesis	▲
Photosynthesis	●	RNA transport	■	Galactose metabolism	★
Alanine, aspartate and glutamate metabolism	▲	Phenylalanine, tyrosine and tryptophan biosynthesis	◆	Glyoxylate and dicarboxylate metabolism	●
Circadian rhythm - plant	▼	Phosphatidylinositolsignaling system	●		

★: L4 vs L0; ◆: L4 vs L13; ▲: L4 vs L28; ●: L0 vs L13; ▼: L0 vs L28; ■: L13 vs L28

pathways played specific roles in response to chilling to 0 °C treated temperature; the RNA transport pathway occurred only in L13 vs L28, implying that the RNA transport was the evident difference from the normal growth to the critical growth temperature; the fatty acid biosynthesis pathway, as well as the steroid biosynthesis pathway, the alanine, aspartate and glutamate metabolism pathway, butanoate metabolism pathway occurred only in L4 vs L28, implying that 4 pathways specifically responded to chilling in the wild banana; and finally, the plant-pathogen interaction pathway and the phenylalanine, tyrosine and tryptophan biosynthesis pathway occurred only in L4 vs L13, implying that 2 specific pathways were the evident difference from the critical growth temperature to the chilling temperature, which might be very important for chilling responses. Some pathways occurred in 2–5 groups, such as the carotenoid biosynthesis pathways in L0 vs L28 and L4 vs L28, implying that the carotenoid biosynthesis was the evident difference of response between the normal growth temperature and the chilling and 0 °C treated temperature; the RNA degradation pathway and the protein export in pathway in L0 vs L28 and L13 vs L28, implying that the 13 °C and 0 °C, but not 4 °C, were likely the 2 main peaks of RNA degradation and protein export; the spliceosome pathway in L4 vs L13 and L0 vs L13, implying that the splicesome played important roles such as alternative splicing at the chilling and 0 °C treated temperatures; the glycerophospholipid metabolism in L4 vs L0, L4 vs L13, L0 vs L13, implying that both for chilling and for 0 °C treated temperature, the glycerophospholipid metabolism changed significantly; the endocytosis pathway in L13 vs L28, L4 vs L13, L4 vs L0 and L0 vs L28, implying that endocytosis occurred significantly during all the 3 cold-stress temperatures; only selenocompound metabolism pathway in 5 groups except for L4 vs L28 and the TCA cycle pathway in 5 groups except for L13 vs L28, implying that selenocompound metabolism and the TCA cycle played

important roles in response to cold stress. Among all the top 20 enriched pathways, only the sulfur metabolism pathway and purine metabolism pathway occurred in all the groups, which suggested that sulfur metabolism and purine metabolism play a vital role in response to cold stress in the banana, and sulfur and purine be also applied to alleviate cold stress.

Moreover, the scatter diagrams of the top 20 KEGG pathways enriched of the putative target genes for DE miRNAs were showed as Additional files 15, 16. Overall, the most enriched pathway evaluated by rich factor was sulfur metabolism pathway, and the second sulfur relay system pathway or selenocompound metabolism pathway; beyond the chilling temperature of 4 °C (L4 vs L13, L4 vs L28 and L13 vs L28), the sesquiterpenoid (including ABA and SL biosynthesis) and triterpenoid biosynthesis pathway shared the most enriched, which suggested that sesquiterpenoid and triterpenoid biosynthesis may play essential roles in response to chilling in the banana. In addition, the circadian rhythm-plant pathway occurred only in L0 vs L28, which implied that circadian rhythm might play special role in response to 0 °C treated temperature in the banana.

Together with the annotations for predicted target genes of DE miRNAs (Additional file 10) and combined with GO and KEGG pathway enrichment analyses, the functions of the candidates of targets for the DE miRNAs in response to the cold stress were described as follows:

The predicted target genes for DE miRNAs during cold stress in the wild banana indicated that there existed diversified cold-responsive pathways, which suggested the miRNAs play critical roles in response to cold stress by regulating ROS (reactive oxygen species) scavenging, DNA repair, activation of the CBF (C-repeat binding transcription factor) pathway, the process related to stomatal closure, keeping osmotic homeostasis, secondary metabolites and the signaling of phytohormones for cold acclimation. Notably, TFs, sulphur (S) metabolism, hydrolysis, protein kinases and secondary metabolism related genes were targeted by so many DE miRNAs, suggesting the specific functions of TFs involved in signal transduction of cold stress through regulating related functional genes, S metabolism for alleviating cold induced oxidative stress by improving proline and glutathione production, hydrolysis for keeping osmotic homeostasis and other cold acclimation, protein kinases involved in signal transduction of MAPK (Mitogen-activated protein kinase) and CDPK (calcium-dependent protein kinase) cascades, and secondary metabolism for taking flavonoids, steroids, carotenoids and anthocyanins and so on, as antioxidants for cold acclimation under cold stress in the wild banana.

### Many target candidates belonged to transcription factors (TFs) genes in the wild banana

Many target candidates belonged to TFs genes, and some of them occurred at high frequencies (Additional file 17), such as the genes for Zinc-binding motif transcription factors (zinc finger), myb, SBP-box, WD40-repeat-containing domain, AP29, bzip, AUX/IAA protein, growth regulating factor (WRC), Basic Helix-loop-helix domain (bHLH), no apical meristem (NAM), Auxin response factor (ARF), Mcm1-Agrmous-Deicicens-SRF4-box transcription factor (MADS box), Gibberelic Acid Insensitive Repressor of ga1–3 Scarecrow, GAI, RGA and SCR (GRAS), etc., with the frequencies > 10, which suggested that these TFs play key roles in regulating expressions of cold-responsive genes during cold stress in the wild banana. It is reported that TFs involved in plant terpenoid biosynthesis are apetala 2 transcription factors (AP2), zinc finger, basic region/leucine zipper motif transcription factors (bzip), Helix-loop-helix domain [66], which ranked ahead at the frequencies in our results, and further supported the results of KEGG pathway enrichment analysis of the predicted targets for the DE miRNAs, i.e., the sesquiterpenoid and triterpenoid biosynthesis pathway was one of the most enriched pathways, and the carotenoid biosynthesis pathway was the evident difference of response between the normal growth temperature and the chilling/0 °C treated temperature, both of which belonged to the terpenoid biosynthesis pathway, and maybe played essential roles in response to chilling; for the second instance, recent studies showed SBP-box TFs, belonging to a specific gene family of plants, are involved in hormone signal transduction and responses to abiotic and biotic stress in many species, and existence of SBP-boxes in this study showed hormone signal transduction might be of importance in response to cold stress; for another instance, myb and WD repeat-containing protein (WD) 40 TF proteins can work individually or orchestrate with other TFs in controlling the multiple enzymatic steps involved in the flavonoid biosynthetic pathways of various species [67, 68], which implied that flavonoid biosynthesis was also a key response to the cold stress in the wild banana.

### The target candidates of the genes coding protein kinases, DUFs, TPRs, ATPases, ribosomal proteins and hydrolytic enzymes occurred at high frequencies in the wild banana

Interestingly, among all the non-TF target candidates, the highest frequency occurred in the genes coding protein kinases (6%), followed by DUF proteins (domain of unknown function), tetratricopeptide-like helical proteins, ATPases and ribosomal proteins, with the frequencies between 48 and 102, and then, glycoside hydrolases, peptidase, P-loop containing nucleoside triphosphate (NTP) hydrolases, armadillo-like helical proteins, with the frequencies between 40 and 43. It thus implied that the biochemical processes related to these enzymes, especially protein kinases and hydrolytic enzymes, were active in response to cold stress in the wild banana. For example, the protein kinases MAPK and CDPK were involved in the cold-stress signal transduction cascades; tetratricopeptide-like helical proteins containing TPRs (tetratricopeptide repeat motif proteins) were involved in a variety of biological processes, e.g., cell cycle regulation, transcriptional control, mitochondrial and peroxisomal protein transport, and protein folding; ATPases and ribosomal proteins function in energy release, protein biosynthesis and DNA repair, respectively; glycoside hydrolases assisted in the hydrolysis of glycosidic bonds in complex sugars, which was helpful for cold acclimation; the most common reaction catalysed by enzymes of the P-loop NTPase fold was the hydrolysis of the beta-gamma phosphate bond of a bound NTP, and the energy from NTP hydrolysis was typically utilized to induce conformational changes in other molecules, which constituted the basis of the biological functions of most P-loop NTPases; β-catenin belonged to the armadillo-like helical protein, the term armadillo derived from the historical name of the β-catenin, and the protein kinase-CK2, which phosphorylated the armadillo repeat region of beta-catenin, and further potentiated Wnt signaling, leading to proteasome resistance and increased protein and co-transcriptional activity in animals. All of which also implied that CK2 likely played an important role in response to cold stress in the wild banana. Furthermore, based on the above analysis, it was found that only 15% were for TFs, which pointed two regulation pathways: i) the miRNAs highly and effectively affected the expression levels of cold-responsive genes by regulating TFs during cold stress; ii) for most of targets, the miRNAs might directly regulate the expression levels of cold-responsive genes that responded to cold stress quickly. On the other hand, the flavonoid biosynthesis and the terpenoid biosynthesis regulated by the related miRNAs via TFs might be slowly responded to cold stress in the wild banana.

### Target candidates of DE miRNAs involved in the sulfur metabolism and sulfur relay system pathways, and the related selenocompound metabolism pathway in the wild banana

The KEGG analysis indicated that the most enriched pathway evaluated by rich factor was sulfur metabolism pathway, and the second was sulfur relay system pathway or selenocompound metabolism pathway. Sulfur metabolism pathway occurred in all the 6 groups at the top 20 enriched pathways, and the most enriched pathway evaluated by rich factor was the same sulfur metabolism pathway compared the L0 library with the other 3 libraries (L4, L13, L28). The sulfur relay system pathway showed the same tendency. It was known that plants had a set of transporters and enzymes that mediated uptake and assimilation of inorganic sulfate and subsequent metabolic conversion to organic sulfur compounds, and sulfate assimilation included uptake and transport, activation, reduction and cys synthesis of sulfate, and sulfate uptake and internal remobilization of sulfur source were generally induced by *sulfur limitation (SLIM) 1* under sulfur deficiency [69]. *SLIM1* was suggested as a key regulator in transcriptional regulation of sulfate transport system, and miR395 negatively controlled *sulfate transporter* and *ATP sulfurylase (ATPS)* and further downstream regulatory cascades [70]. MiR395 was specific for its accumulation under low-sulfur conditions. MiR395 had a complementary sequence that hybridized with *low-affinity sulfate transporter SULTR2;1* and three isoforms of *ATPS*, *ATPS1*, *ATPS3*, and *ATPS4* [71–73]. MiR395 was strongly up-regulated by sulfate deficiency and targeted two components of the sulfate uptake and assimilation pathway, i.e., *sulfate transporter* and *ATPS*, which were suggested to be an integral part of the regulatory network of sulfate assimilation [74]. There existed interplay of *SLIM1* and miR395 in the regulation of sulfate assimilation in *Arabidopsis* [75]. Selenium (Se) accumulation was involved in sulfur assimilation, antioxidant activities and defense genes of jasmonic acid and salicylic acid pathway [76], and Se induced S deficiency to promote sulfate assimilation for producing more H_2_O_2_ to control ROS levels. Many cofactors and nucleotides containing sulfur atoms were known to have diversified important functions. Recently, the biosynthetic pathways of these sulfur-containing compounds were revealed, where many enzymes relayed on sulfur atoms [77]. Here, we found that in L4 vs L0, there were target candidates for DE miRNAs as follows: *sulfate adenylyltransferase* had 3 members, the same as *adenylyl-sulfate kinase*, which catalyzed sulfate to adenosine-5′-phosphosulfate (APS) and 3′-phosphoadenosine-5′-phosphosulfate (PAPS), respectively, and they belonged to the genes of sulphate adenylyltransferase with PUA-like domains, which negatively regulated by miR395 (Additional file 18-a); *thiosulphate sulfurtransferase* (*Tum1*) had 2 members targeted by miR171, which catalyzed thiosulphate to sulphate; The gene coding EC1.8.7.1 had only one target candidate (Nitrite/sulphite reductase 4Fe-4S domain /SIR) targeted by miR172; The gene coding EC 2.3.1.30 also had 1 target; cysteine synthase had 2 members-one targeted by gma-miR6300 and the other targeted by osa-miR827, which catalyzed sulphate to L-cysteine to link the pathway of cysteine and methionine metabolism. MiR395, miR827, miR171 and miR172 were upregulated at 4 °C and downregulated at 13 °C, whereas miR6300 downregulated at 4 °C. In the sulfur relay system pathway, the target candidate *Tum1* for DE miRNAs had 2 members-one targeted by ata-miR395b-3p and the other targeted by miR171; the *ubiquitin-related modifier 1* (*Urm1*) had only one member targeted by miR399, which were significantly upregulated at 4 °C or 0 °C (Additional file 18-b), and the 3 miRNAs (miR395, miR171 and miR399) seemed to be of great importance for chilling and 0 °C treated response to cold stress. Selenate was chemically similar to sulfate and was taken up and assimilated by plants via the same transporters and enzymes. Interestingly, among all the groups, selenocompound metabolism pathway, the gene coding EC 2.7.7.4 target (*ATP-sulfurylase*) for DE miRNA had 3 members, which catalyzed selenate to adenosine 5′-phosphoselenate (APSe), and all of which were also targeted by miR395 as the sulfate transport system did in the sulfur metabolism (Additional file 18-a). To catalyze selenate to APSe, or to activate selenate in the reduction of selenate, was the first step to utilize Se in plants. Therefore, it was inferred that miR395 was not only a key integral part in the regulatory network of sulphate assimilation and sulfur relay system, but also in the regulatory network of selenate assimilation.

### Target candidates of DE miRNAs involved in the sesquiterpenoid and triterpenoid biosynthesis pathway in the wild banana

Gene annotation of the wild banana indicated that there were 2 branches at the node of Farnesyl PP farnesyl pyrophosphate (FPP) (also known as farnesyl diphosphate, FDP) in the sesquiterpenoid and triterpenoid biosynthesis pathway: one was the pathway branch of sesquiterpenoid synthesis via multiple procedures, including synthesis of acyclic sesquiterpenoids in which (E, E) farnesol was catalyzed by NAD^+^-dependent farnesol dehydrogenase/FLDH (the gene coding EC 1.1.1.354) to form farnesal; the other was from FPP to synthesize presqualene-PP and further squalene catalysed by farnesyl-diphosphate farnesyltransferase (the gene coding EC 2.5.1.21/FDFT1), and squalene was catalyzed by squalene monooxygenase /SQLE, ERG1 (the gene coding EC 1.14.14.17) to form (S)-squalene-2,3-epoxide to link steroid biosynthesis pathway. Squalene and (S)-squalene-2,3-epoxide connected to chair-chair-chair-chair confirmation and chair-chair -chair-boat confirmation biosynthesis pathways, respectively.

During cold stress in the wild banana, the sesquiterpenoid and triterpenoid biosynthesis pathway shared the most enriched beyond the chilling temperature of 4 °C (L4 vs L13, L4 vs L28 and L13 vs L28), and the gene coding EC 1.14.14.17 target of DE miRNA had only one member squalene epoxidase (SQLE) targeted by cca-miR396a-3p, which was significantly upregulated at 4 °C. It had 5 members with the highest enriched factor in L4 vs L13, except for 1 member targeted by cca-miR396a-3p, all the others were targeted by vvi-miR172a, which was significantly and specifically upregulated at 4 °C. Again, it had 1 member in L13 vs L28, targeted by cca-miR396a-3p and the gene coding EC 2.5.1.21 was also predicted in the group, which had one member targeted by cca-miR156b and catalysed the synthesis of presqualene-PP and squalene (Additional file 18-c).

### Target candidates of DE miRNAs involved in carotenoid biosynthesis and flavonoid biosynthesis pathways in the wild banana

According to KEGG annotations, the target candidates for miRNAs involved in carotenoid biosynthesis pathway in the wild banana were as follows: i) Phytene was synthesized from geranylgeranyl pyrophosphate (GGPP) catalyzed by phytoene synthase (the gene coding EC 2.5.1.32/ CrtB), and then there were 2 pathways to form lycopene from phytene: one was the pathway that lycopene was formed via catalyzing a searies of enzymes including phytoene desaturase (PDS), 15-cis-zeta-carotene isomerase (Z-ISO), zeta-carotene desaturase (ZDS) and carotene isomerase (Crt-ISO); the other was the pathways that phytofluene and ζ-carotene was first formed catalyzed by PDS, and then ζ-carotene was catalyzed by ZDS to form neuroporene, and finally lycopene was formed from neuroporene catalyzed by ZDS. There was no branch in the first pathway, but there was a branch in the other pathway, i.e., β-zeacarotene and 7,8-dihydro-β-carotene, were formed from neuroporene catalysed by carotene lycopene β-cyclase (CrtL-b). ii) The downstream products, such as δ-carotenoid, ε-carotenoid, α-carotenoid, β-carotenoid, γ-carotenoid, 9-cis-β-carotenoid, 9-cis-10-Apo-β-carotenal, carlactone, α-crytoxanthin, zeinoxanthin, lutein (xanthophyll), zeaxanthin, β-crytoxanthin, anthelaxanthin, 9-cis-violaxanthin, violaxanthin, neoxanthin, xanthoxin, asataxanthin, and other downstream products, were formed from several downstream pathways, i.e., one was the pathway that δ-carotenoid, ε-carotenoid and α-carotenoid formed catalyzed by CrtL-eand CrtL-b, from lycopene and further α-crytoxanthin was formed catalyzed by Lutein 1 (LUT1) from α-carotenoid, and then from which lutein was formed catalyzed by Crt-b, or zeinoxanthin was formed catalyzed by LUT5/Crt-b from α-carotenoid, and then from which lutein was formed; the other was that γ-carotene was formed firstly catalyzed by Crt-b from lycopene, and then β-carotene was further formed catalyzed by Crt-b. β-carotene was linked to 3 downstream biosynthesis pathways: i) via β-crytoxanthin and zeaxanthin to link to asataxanthin biosynthesis pathway; ii) via 9-cis-β-caroteoid, 9-cis-10-Apo-β-carotenal and carlactone forming to link to strigolactone (SL) biosynthesis pathway (carlactone was the precursor of SL biosynthesis); iii) via β-crytoxanthin, zeaxanthin, anthelaxanthin, 9-cis-violaxanthin, violaxanthin, neoxanthin and xanthoxin forming to link to ABA biosynthesis pathway (xanthoxinis the precursor of ABA biosynthesis).

In the scatter diagrams of the top 20 enriched pathways of the targets of DE miRNAs, the carotenoid biosynthesis pathway occurred only in the 2 groups (L0 vs L28 and L4 vs 28), which indicated that it existed in the chilling (4 °C) and 0 °C treated temperature (0 °C) and played an important role in response to chilling and 0 °C treated temperature. However, there was no significant effect under the critical growth temperature of 13 °C. The KEGG figures indicated that synthetases related to the major carotenoids were differentially expressed in the carotenoid biosynthesis pathway, and the synthetic major carotenoids included phytene, phytofluene, lycopene, ζ-carotene, β-zeacarotene and 7,8-dihydro-β-carotene, δ-carotenoid, ε-carotenoid, α-carotenoid, β-carotenoid, γ-carotenoid, 9-cis-β-carotenoid, 9-cis-10-Apo-β-carotenal, carlactone, α-crytoxanthin, zeinoxanthin, lutein (xanthophyll), zeaxanthin, β-crytoxanthin, anthelaxanthin, 9-cis-violaxanthin, violaxanthin, neoxanthin, xanthoxin, asataxanthin, and so on, which contained the most components of carotenoids in the plants. Interestingly, β-zeacarotene and 7,8-dihydro-β-carotene, of which the synthetic pathway was different from the others and they formed directly from neuroporene but not from lycopene. In the previous reports, the β-zeacarotene and 7,8-dihydro-β-carotene have been less paid attention to, but the related miRNAs were differentially expressed, suggesting the importance of the pathway branch in the response to cold stress in the wild banana. In the meanwhile, the carotenoid pathway was linked to asataxanthin and ABA biosynthesis pathways, indicating that both asataxanthin and ABA were also involved in the response to cold stress in the wild banana. It was reported that astaxanthin showed both a strong quenching effect against singlet oxygen, and a strong scavenging effect against free radicals, and the activities of astaxanthin were approximately 10 times stronger than those of the other carotenoids that were tested, namely zeaxanthin, lutein, tunaxanthin, canthaxanthin and β-carotene, and 100 times greater than those of a tocopherol. Astaxanthin also showed strong activity as an inhibitor of lipid peroxidation mediated by these active forms of oxygen [78]; and the ABA-dependent pathway for cold acclimation was very essential for plant cold-resistance. In addition, the carotenoid biosyntesis pathway in the wild banana was found to linked to SL biosyntesis pathway, suggested that the SLs might be involved in response to cold stress in the wild banana [79, 80]. In a word, carotenoids, especially astaxanthin, ABA and SL likely played important roles in the response to cold stress and in the cold acclimation in the wild banana.

Most of the target candidates for DE miRNAs coded the upstream enzymes, e.g., *CrtL-e* (*lycopene cyclase-type*) targeted by stu-miR172c-3p, ath-miR172a and aly-miR172e-3p; *ZDS* targeted by sa-miR166g-3p, osa-miR166e-3p, ptc-miR166n, vvi-miR166a, cme-miR166i; *PDS* targeted by osa-miR5532, and all the miRNAs of the 3 targets expressed differentially during cold stress in the wild banana. In addition, one more target was predicted in L4 vs L28, which was *Crt-ISO* targeted by vvi-miR156h. The vvi-miR156h was up-regulated at 4 °C but down-regulated at 0 °C and 28 °C (Additional file 18-d), which suggested that the first pathway branch forming lycopene from phytene should be blocked under the chilling temperature, and the other pathway branch should be enhanced, resulting in an increase of β-zeacarotene and 7,8-dihydro-β-carotene accumulation in the biosynthesis pathway, which further implied that the 2 kinds of carotenoids might play a key role in cold acclimation in the wild banana. Francis et al. (1996) [81] reported that β-zeacarotene could further form β-carotenoid. Few studies on the antioxidant activities of β-zeacarotene and 7,8-dihydro-β-carotene were reported, and there were almost no reports on the relationship between the carotenoids of β-zeacarotene and 7,8-dihydro-β-carotene and the cold stress. It was worthwhile to further exploring the relations.

The flavonoid biosynthesis pathways were enriched in the 4 groups (L0 vs L4, L0 vs L13, L4 vs L28, L13 vs L28). There were 2 targets in L4 vs L28, *oxoglutarate/iron-dependent dioxygenase* targeted by vvi-miR156h, and the other targeted by osa-miR5538; and other groups had only one target. The L4 vs L0 had the target *oxoglutarate/iron-dependent dioxygenase* targeted by bdi-miR845; the L0 vs L13 had the target *NAD-dependent epimerase/dehydratase* taregted by osa-miR5538; and the L13 vs L28 had the one targeted by osa-miR5538. The osa-miR5538 occurred in L0 vs L13, L4 vs L28, L13 vs L28, and was upregualted in the 13 °C and 4 °C, implying that it was responsive to chilling stress but not 0 °C treated stress. The bdi-miR845 was specifically up-regulated in 0 °C but down-regulated in all the other tested temperatures, which might be specifically responsive to 0 °C treated stress (Additional file 18-e).

From the above analyses of the targets candidates of DE miRNAs, it was concluded that the miR172, miR156 and miR166 might be involved in the carotenoid biosynthesis and osa-miR5538 and miR845 in the flavonoid biosynthesis pathways during cold stress in the wild banana.

### Target candidates of DE miRNAs involved in the circadian rhythm in the wild banana

The circadian clock is an endogenous mechanism that coordinates biological processes with daily and seasonal changes in the environment. In *Arabidopsis*, three genes were found to code the core components of the central oscillator: circadian clock associated 1 (CCA1), late elongated hypocotyl (LHY), and timing of CAB expression 1 (TOC1). CCA1 and LHY bound directly to the promoter of *TOC1*, negatively regulating *TOC1* expression, and *TOC1* participated in the positive regulation of *CCA1* and *LHY* expression by an unknown mechanism [82–84]. Others, such as phytoclock 1, gigantean (GI), early flowering 3 (ELF3), ELF4, time for coffee, and pseudo response regulator (PRR) 3/5/7/9, had also been suggested to function in or close to the central oscillator [85–87], among of which GI was suggested as a remote regulating factor. After translation, the CCA1 protein needed to be phosphorylated by CK2, and the phosphorylation is necessary for the protein to form a homodimer and to bind to its target promoters, which suggested that CCA1 phosphorylation by CK2 was important for the normal functioning of the central oscillator [88]. Constitutively photomorphogenic 1 (COP1), containing a ring finger zinc-binding motif, a coiled-coil domain, and several WD-40 repeats, similar to G-beta proteins, was a ring-finger-type ubiquitin E3 ligase, which functions downstream of cryptochrome and phototropin photoreceptors to repress stomatal opening, and which was also a known suppressor of photomorphogenesis, acting through ubiquitylation and targeted degradation of several light-signaling factors including hypocotyl 5 (HY5) [89–91]. It had been demonstrated that suppressor of phytochrome A 1 (SPA1) was associated with COP1 to promote COP1 activity and suppressed photomorphogenesis [92]. In the meanwhile, COP1 also regulated temperature sensitivity by controlling the degradation of GI, and further to remotely regulates CCA1 through GI [93]. Recently, it was reported that HY5 was also important for the coordination of nitrogen (N) and sulfur assimilation. HY5 could be considered as a new regulator of the pathway, and as a link between N and sulfate assimilation. HY5 was involved in the regulation of adenosine 5′-phosphosulfate reductase (APR) gene expression, and the key enzyme of sulfate assimilation, adenosine 5′-phosphosulfate reductase, was regulated by HY5 in *Arabidopsis* [94, 95]. HY5 also regulated nitrite reductase 1 (NIR1) and ammonium a transporter (AMT) 1;2 in *Arabidopsis* seedlings [96].

**Fig. 5:**
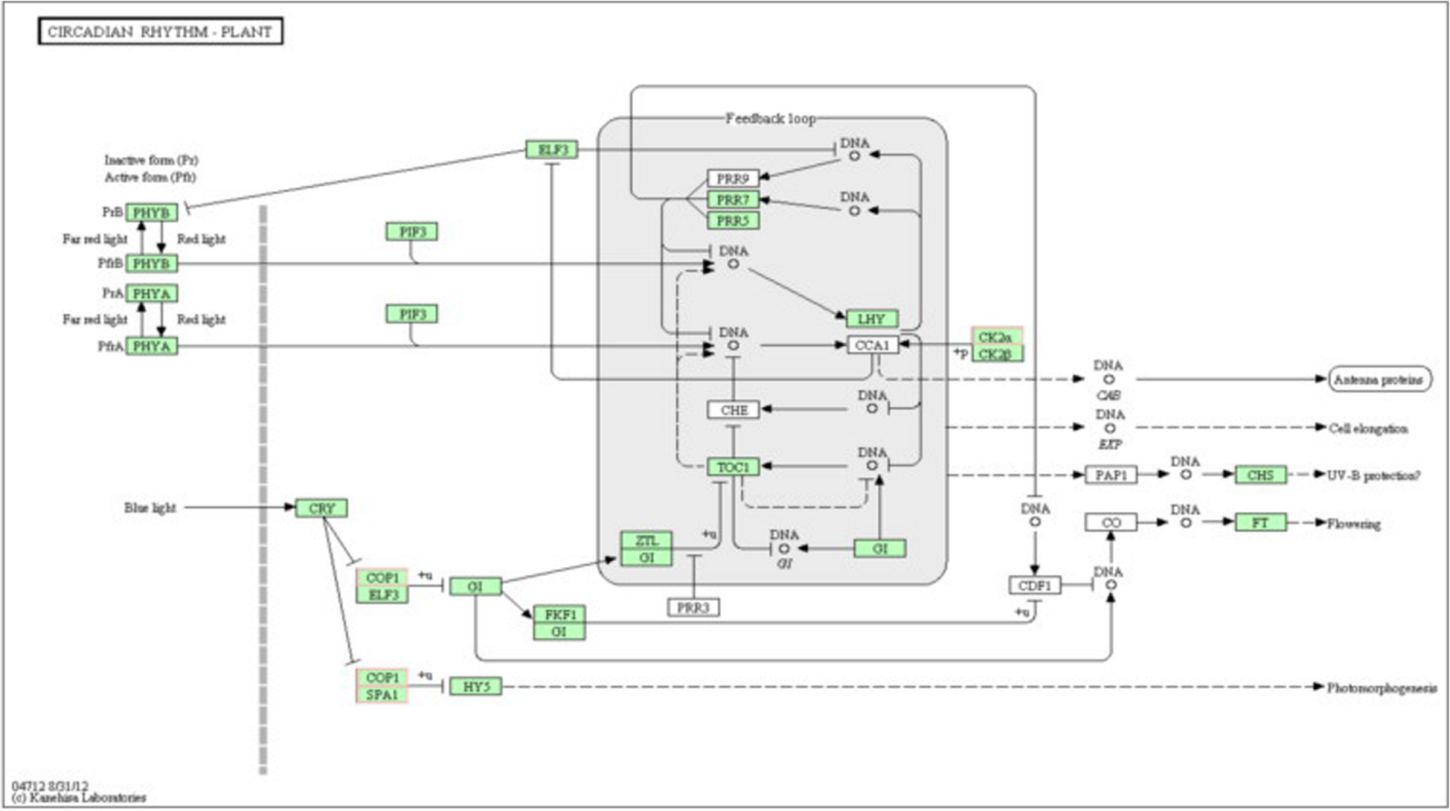
The plant circadian rhythm of the top 20 KEGG pathways in L_0 vs L28. The map was provided permission from Kanehisa laboratory [178–180]. The nodes are marked in red background color indicating the DEGs is up-regulated expression, and the nodes are marked in green background color indicating the DEGs is down-regulated expression. On the other side, the white nodes indicated the genes are DEGs, but there is no distinguishment of the DEGs about the up- / down-regulated expression

In the wild banana, the circadian rhythm-plant pathway shared the top 20 pathways in L0 vs L28 (Fig. 5), which indicated that the circadian rhythm pathway was specific and essential to 0 °C treated response to cold stress. The targets of DE miRNAs included the genes coding CK2α, COP1 and SPA1, all of them were of protein kinases and could be individed into 2 categories: one was CK2α, serine/threonine−/ dual-specificity protein kinases with ATP binding site, which had 4 members, and all of them were targeted by stu-miR172c-3p; and the other was the protein kinases with WD40/YVTN repeat-like-containing domain, including COP1 and SPA1. *COP1* was targeted by lus-miR172j and *SPA1* was targeted by ath-miR168a-5p and gma-miR168b. *CK2*, belonging to the dual specificity protein kinase, was involved in stress-activated MAPK cascade, implying the 4 members of *CK2* might activate the MAPK cascades in response to 0 °C treated temperature in the wild banana. In a word, the 3 targets of DE miRNAs in the circadian rhythm-plant pathway, i.e., the genes coding *CK2*, *COP1* and *SPA1*, were the key components responsive to 0 °C treated stress in the wild banana, and it was inferred that during 0 °C treated stress the key protein kinases of *CK2* and *COP1* regulated biological clock to synchronize related signaling pathways and further activate various cold acclimation pathways; in the meanwhile, COP1/SPA1 complex negatively regulated HY5 to alleviate growth of plants for cold acclimation by the regulation of sulfur and N assimilation by HY5. Furthermore, miR172 regulated *CK2* (by miR172c) and *COP1* (by miR172j) to control biological clock. Therefore, miR172 family was the key miRNA for regulating biological clock during cold stress in the wild banana.

### Target candidates in other metabolic pathways in the wild banana

Combined with the above analyses, miR172c regulated several essential pathways or biological processes during cold responses in the wild banana, such as sulfur metabolism pathway, lycopene cyclase, histone acetylation/deacetylation, circadian rhythm-plant pathway, etc. Thus, we thought that the miR172 family might play essential roles in cold responses in the wild banana, and the analyses of the targets of miR172 family were further conducted as follows:

There were 10 members of miR172 family in the wild banana, i.e., miR172a, miR172b, miR172b-5p, miR172c, miR172c-3p, miR172d, miR172e, miR172e-3p, miR172f, miR172j. The total 1060 target candidates of miR172 family were mapped to the Banana Genome, including 139 target candidates for stu-miR172c-3p, 114 target candidates for aly-miR172e-3p, 86 target candidates for gma-miR172b-5p, 85 target candidates for sbi-miR172b, 79 target candidates for ath-miR172a, 75 target candidates for vvi-miR172a, 75, 72 target candidates for vvi-miR172a and vvi-miR172b, 66 target candidates for ath-miR172e-3p, 65 target candidates for osa-miR172c, 61 target candidates for ath-miR172c, 53 target candidates for lus-miR172j, 51 target candidates for cme-miR172f, 41 target candidates for mtr-miR172a, 38 target candidates for mes-miR172c, 35 target candidates for gma-miR172d, among of which stu-miR172c-3p targeted the most candidate genes, including the genes coding AP2/ARF domains, serine/threonine−/dual-specificity protein kinase, ATP binding site, uroporphyrin-III C-methyltransferase, WD40/YVTN repeat-like-containing domain (related to ubiquitylation), adaptor 1 (ada)/transcriptional adaptor 1 (tada) (related to histone acetylation), S-adenosyl-L-methionine-dependent methyltransferase mida, zinc finger (Ring/Fyve/Phd-type) (TFs, related to auxin signalling), p-loop containing NTP hydrolase, actin-related protein 4 (Arp4) (related to histone acetylation/chromatin remodeling), C2 calcium-dependent membrane targeting, calmodulin binding protein-like, CRC domain, cytochrome b5, glutathione S-transferase, glycoside hydrolase, heat shock protein dnaj, histone deacetylase interacting with paired amphipathic helix (related to histone deacetylation), histone H4, myb, kinesin, lycopene cyclase, beta/epsilon, PTR, WW/Rsp5/WWP, PX-associated, sorting nexin 13, hydroxymethylglutarylcoenzyme A synthase, phenylalanyl-tRNA synthetase, ubia prenyltransferase family, vacuolar protein sorting-associated protein 62, zinc fingers (related to auxin signalling), sodium/sulphate symporter, snf 7, histone acetyltransferase ELP3, NB-ARC, NAD-dependent epimerase/dehydratase, armadillo-like helical, glucose/ribitol dehydrogenase, and so on, which suggested that stu-miR172c-3p can simultaneously regulated phosphorylation of CK2, histone methylation, secondary metabolites methylation, histone acetylation/deacetylation, ubiquitylation, chromatin remodeling, ATP energy conversion, microtubule movement, DNA/RNA uncoiling, thylakoid protein transport, protein sorting, sulfur metabolism, AP2/ARF proteins, etc., and which showed that miR172c-3p played various roles and coordinated many special key biological processes in responses to cold stress in the wild banana. Besides, the other members of miR172 family could also coordinately regulate many biological processes.

The biological clock regulated many physiological and biochemical processes in plants. For example, it was reported that the circadian clock regulated the fluxes of solutes, including water fluxes, ions such as K^+^, metabolic solutes such as sucrose, essential nutrients such as N and S, and signaling molecules calcium, etc. [97], and the rhythmic metabolism contributed to the circadian network, which included photosynthesis, starch metabolism, nutrient assimilation and redox homeostasis in plants [98]. In *Arabidopsis*, transcriptome experiments demonstrated circadian regulation of key transcripts involved in photosynthesis and starch metabolism, phenylpropanoid (flavanoid and anthocyanin) biosynthesis, isoprenoid (chlorophyll and carotene) biosynthesis and redox balance [82, 99]. In our analysis, miR172 family was suggested to be the key miRNA for regulating biological clock (targeting *CK2*, *COP1* and *SPA1*) during cold stress in the wild banana, and was also predicted to target genes related to phosphorylation of CK2, histone methylation, secondary metabolites methylation, histone acetylation/deacetylation, ubiquitylation, chromatin remodeling, ATP energy conversion, microtubule movement, DNA/RNA uncoiling, thylakoid protein transport, protein sorting, sulfur metabolism, AP2/ARF proteins, etc. Therefore, it was further certified that miR172 family (especially miR172c) should play a central coordinating role in regulating biological clock during cold stress in the wild banana.

Some essential target candidates of DE miRNAs in other pathways in the wild banana were presented as follows:

i) The fructose and mannose metabolism pathway shared the top 20 pathways only in L0 vs L13 (Additional file 18-f). It indicated that the fructose and mannose metabolism was involved in the response to 0 °C treated stress. There were 2 targets, i.e., GDP-L-fucose synthase with 2 members and phosphorfructokinase with 1 member, both of which were targeted by bra-miR408-5p, and were significantly up-regulated at 4 °C and down-regulated at 0 °C. GDP-L-fucose synthase catalyzed GDP-4-dehydro-6-deoxy-D-mannose to form GDP-L-fucose, and the reaction is reversible, and both of the GDP-D-mannose and GDP-L-fucose were important nucleotide sugars and essential substrates for glycosylphosphatidylinositol (GPI) anchor biosynthesis and synthesis of the anchors for N-glycans (N-glycans biosynthesis); while EC 2.7.1.11 was a kinase enzyme that and catalyzed the phosphorylation of fructose-6-phosphate to form fructose-1,6-bisphosphate, which was a key regulatory step in the glycolytic pathway and the kinase was also the rate-limiting enzyme in glycolysis. Therefore, the DE miR408 might be essential for sugar-related cold acclimation during cold stress in the wild banana.

ii) The other glycan degradation pathway shared the top 20 KEGG pathways only in L4 vs L28; L13 vs L28 and L4 vs L0 (Additional files 14, 15), of which the most enriched was in L4 vs L28, indicated that the glycan degradation pathway was involved in the response to chilling and 0 °C treated temperature. In L4 vs L28, there were 4 targets, i.e., *glycoside hydrolase* targeted by gma-miR172b-5p, which was significantly and specifically up-regulated at 4 °C; *glycosylhydrolase* targeted by gma-miR6300 and bdi-miR845, and the former was significantly down-regulated at 4 °C but the latter was significantly and specifically up-regulated at 0 °C; *glycoside hydrolase* (*DUF640*) targeted by gma-miR5368, which was significantly and specifically up-regulated at 28 °C; *beta-glucosidase* (*GBA2 type*) targeted by osa-miR156l-5p, zma-miR156k-5p and cca-miR156b, the former two of which were significantly and specifically up-regulated at 4 °C and the last one at 28 °C. In a word, 1–4 targets of DE miRNAs occurred at different groups; interestingly, the DE miRNAs were generally up- or down-regulated at 0 °C, 4 °C or 28 °C, suggested that they (bdi-miR845; gma-miR172b-5, psa-miR156l-5p and zma-miR156k-5p; gma-miR5368) were specifically responsive to 0 °C treated, chilling and cold stress, respectively.

iii) Both fatty acid biosynthesis and degradation pathways shared the top 20 pathways only in L4 vs L28 and L4 vs L0, respectively (Additional file 18-g, h). In the fatty acid biosynthesis pathway, there were 3 targets: one was *acetyl-coenzyme A carboxyltransferase*, with 3 members, i.e., GSMUA_Achr4G11690_001 targeted by stu-miR530, GSMUA_Achr8G24640_001 targeted by osa-miR444a-3p.2 and osa-miR444f, GSMUA_Achr10G19000_001 targeted by bdi-miR845, vvi-miR396a, mdm-miR396a and smo-miR396, most of which were up-regulated during cold stress except for vvi-miR396a, and EC 6.4.1.2 catalyzed the irreversible carboxylation of acetyl-CoA to produce malonyl-CoA to provide the substrate for the biosynthesis of fatty acids, which was the first step to link to TCA cycle and fatty acid biosynthesis pathway, and therefore it was the rate-limiting enzyme in biosynthesis of fatty acids; the second was *FabF*, with 1 member, targeted by vvi-miR156h; and the third was EC 6.2.1.3, with 1 member, catalyzed hexadecanoic acid to produce hexadecanoyl CoA, and it linked with *fatty acid desaturase* (*FAD*), targeted by aqc-miR167, which was significantly up-regulated at 4 °C. In the fatty acid degradation pathway, there were 2 targets: one was *AMP-dependent synthetase/ligase*, the same as in the biosynthesis pathway, with 2 members targeted by mes-miR397, linked with the fatty acid biosynthesis pathway; and other was *Acyl-CoA dehydrogenase/oxidase*, with 1 member catalyzes hexadecanoyl CoA to produce *trans-hexadec-2-enoyl-CoA*, targeted by gma-miR168b and ath-miR168a-5p, which were respectively up- or down-regulated at 0 °C and 13 °C, 4 °C, suggesting that they should be specifically responsive to chilling in the wild banana.

In a word, there were some key enzymes as targets of DE miRNAs in the fatty acid biosynthesis and degradation pathways, and the miR845 and miR397 might be the key miRNAs for biosynthesis and degradation of fatty acids, respectively, during cold stress in the wild banana.

iv) The splicesome pathway shared the top 20 pathways only in L4 vs L13 and L0 vs L13, indicating that the splicesome pathway was involved in the response to chilling and 0 °C treated stress, and there were 10 targets in L4 vs L13, *hnRNPs* (*paraneoplastic encephalom yelitis antigen*), targeted by miR319, which was almost significantly down-regulated at 13 °C and up-regulated at 4 °C; *CA150* (*WW/Rsp5/WWP*), targeted by miR156, which was significantly up-regulated at 4 °C; *U2AF* (*U2 auxiliary factor small subunit*), targeted by miR156, and some members were significantly down-regulated in 13 °C and others were significantly up-regulated at 4 °C; *SF3b* (*cleavage/polyadenylation specificity factor*, *A subunit*, *C-terminal*), targeted by bra-miR408-5p, which was significantly up-regulated at 4 °C but significantly down-regulated at 0 °C; the gene coding THOC (THO complex, subunit THOC1), targeted by ahy-miR156a and osa-miR156k, which were up-regulated at 0 °C and 4 °C; *Lsm* (*ribonucleoprotein LSM domain*, eukaryotic/archaea-type) targeted by ata-miR395b-3p, which was significantly up-regulated at 4 °C and down-regulated at 13 °C; *prp22*, targeted by bra-miR408-5p, which was significantly up-regulated at 4 °C and down-regulated at 0 °C; *SR*, targeted by osa-miR156k, vvi-miR156h and ahy-miR156a, all of which were significantly up-regulated at 4 °C; prp43, targeted by gma-miR1511, rgl-miR5139 and cpa-miR8155, which were significantly up-regulated at 0 °C; *CBP80/20*, targeted by cpa-miR8155and rgl-miR5139, which were significantly up-regulated at 28 °C.

However, there were only 5 targets in L0 vs L13, the 5 targets disappeared and the other 5 targets (prp22, hnRNPs, prp43, SF3b, Lsm) remained, indicated that the 5 targets were involved in the chilling and 0 °C treated responses. The 5 targets were responsible for mRNA splicing and alternative splicing, such as prp22 and splicesome components regulate chromatin dynamics [100]; the ATPase activity of prp43 was required for early steps of pre-rRNA processing and normal accumulation of mature rRNAs [101]; SF3B1 as a principal player in the spliceosome and as a target of inhibitor compounds in pre-mRNA splicing complex [102]; hnRNPs are complexes of RNA and protein present in the cell nucleus during gene transcription and subsequent post-transcriptional modification of the newly synthesized RNA (pre-mRNA), and the proteins involved in the hnRNP complexes were collectively known as heterogeneous rib nucleoproteins, which was regulated by phosphorylation catalyzed by protein kinase A and was responsible for suppressing RNA splicing at a particular exon by blocking access of the spliceosome to the polypyrimidine tract [103]. Lsm plays a large number of various roles in mRNA processing and regulation, and depending on which lsm proteins and RNA molecule were involved, this ribonucleic protein complex facilitated a wide variety of RNA processing including degradation, editing, splicing, and regulation [104]. From the above analyses, it was inferred that alternative splicing might play important roles in response to chilling and 0 °C treated temperature via the regulation of miR156, miR319 and miR408 in the wild banana. Interestingly, miR408 was involved not only in sugar-related metabolism, but also in alternative splicing.

v) The related targets of DE miRNAs in some other essential top 20 pathways such as the citrate cycle (TCA cycle) pathway in 5 groups except for L13 vs L28, cysteine and methionine metabolism, peroxisome and endocytosis pathways in 4 groups, carbon metabolism pathway in 3 groups (L4 vs L13, L4 vs L28, L0 vs L13), oxidative phosphorylation pathway in 2 groups (L4 vs L0 and L0 vs L13), the photosynthesis pathway and phosphatidy-linositol signaling system only in L0 vs L13, and so on, were predicted (Additional file 18-i), implying that they might also play important roles in response to cold stress in the banana. Interestingly, most of the key genes were annotated in the phosphatidylinositol signaling system pathway, such as the genes related to the enzymes of PI4K, PIP5K, PTEN, PIKFYVE, PIK3C3, PLC, EC 3.1.3.25, EC 3.1.3.57, EC 3.1.3.64, EC 2.7.1.107, EC 2.7.1.140, EC 2.7.1.151, EC 2.7.1.158, EC 2.7.1.159, EC 2.7.4.24, EC 2.7.7.41, EC 2.7.8.11, and CALM, etc., which was similar to those in human. The transient receptor potential melastatin 8 (TRPM8) ion channel was a physiological sensor of environmental cold temperatures and in human, and TRPM8 was activated through interaction with PIP2 catalyzing by PI4K and PIP5K [105], and though PLC to further link Ca^2+^ signal pathway. Recent studies revealed that there was the similar sensing system in plants [106]. During cold stress in the wild banana, the pathway ranked the top 20 only in L0 vs L13, which there were 2 targets of DE miRNAs-*diacylglycerol kinase* targeted by rgl-miR5139, vvi-miR156h, gma-miR1511, cpa-miR8155, ppe-miR1511–3p and *phosphatidate cytidylyltransferase* targeted by ptc-miR319e, mdm-miR159a, ppt-miR319a, aqc-miR159, ath-miR319c, mtr-miR319a-3p, pta-miR159a, pta-miR319, osa-miR 159c, ath-miR319a; however, the 2 DE miRNA targets of diacylglycerol kinase and the gene coding EC 2.7.7.41 existed among all the 6 groups. Furthermore, the targets of DE miRNAs-*PI4K* targeted by vvi-miR156h, pab-miR3711 and ghr-miR156c and PIP5K targeted by mtr-miR156g-5p, were respectively mapped in L4 vs L28 and L13 vs L28, implying that PI4K and PIP5K were essential for cold sensing and both diacylglycerol kinase and phosphatidate cytidylyltransferase were key enzymes for cold messenger transduction and linking the Ca^2+^ signal pathway during cold stress in the wild banana, which suggested that the phosphatidylinositol signaling system should likely play important roles in response to cold stress in the banana.

In addition, some important target candidates of DE miRNAs in the other pathways were also predicted in one or several groups, which were related to cold responses in plants and might play roles in the responses to cold stress in the wild banana, such as the casparian strip domain-like gene *CASPL*, the genes for the enzymatic antioxidant system, the related genes of Pi starvation, sugar transporting and proline synthesis (sugar transporter, major facilitator superfamily domain targeted by miR3711, *SWEET*, *VIN1* targeted by miR398, prolyl oligopeptidase etc.), SNF1-related protein kinase catalytic subunit alpha 10/11 (KIN10/11), serine hydroxymethyltrans-ferase (SHMT) for photorespiration, C4-related genes, PPP pathway related genes, the genes related to DNA repair complex, ABA biosynthesis pathway and ET biosynthesis pathway, NAD(P)H oxidases, the aspartic acid-glutamic acid-leucine-leucine-alanine (DELLA) for inhibiting the GA response to growth, HO and Nir for promoting stomatal closure, FAD for production of unsaturated fatty acids, kinsesins for regulating assembly/disassembly of cytoskeleton-plasma membrane-cell wall continuums and so on.

### Quantitative PCR analyses of the miRNAs and their target genes in the wild banana

**Fig. 6:**
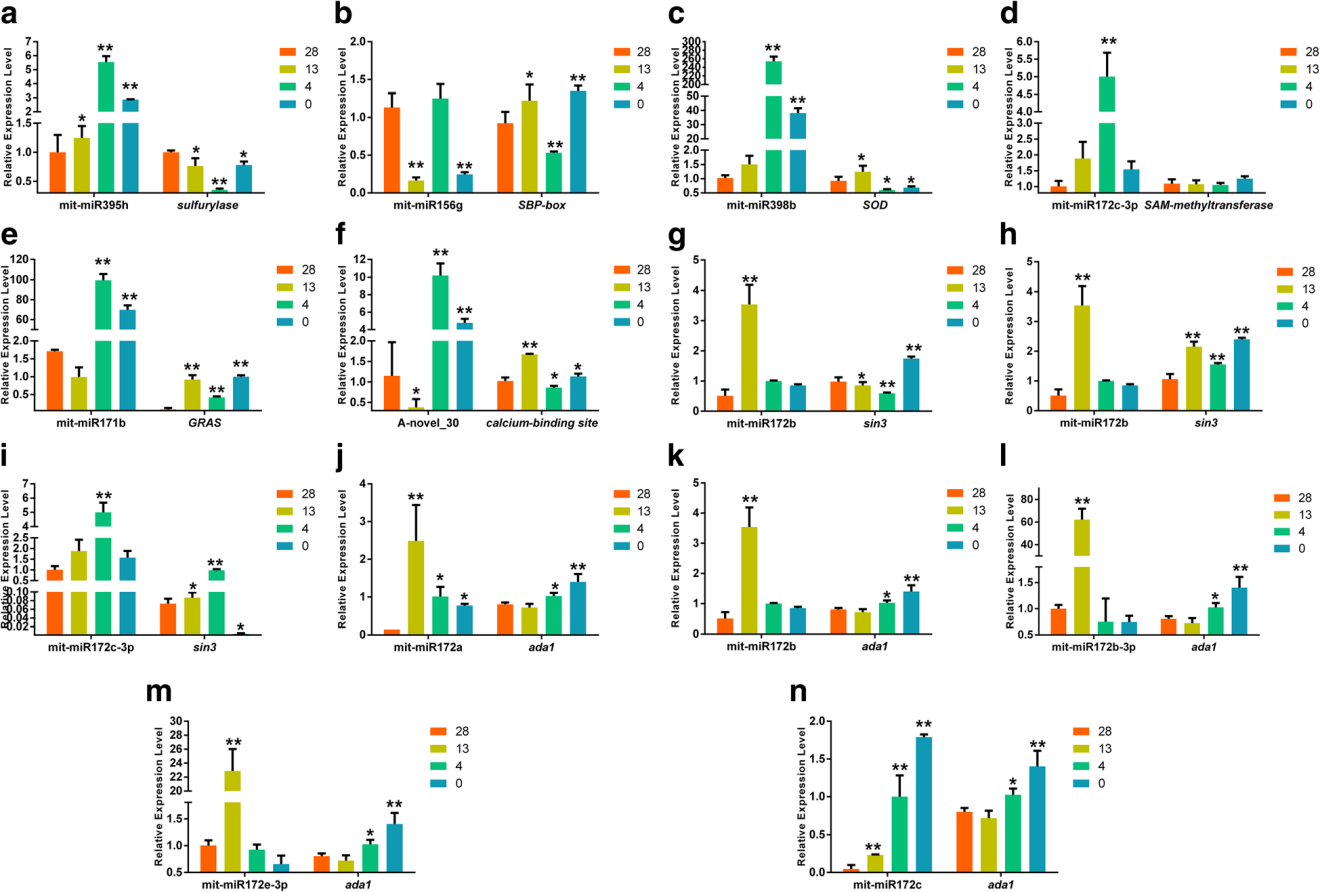
The qPCR validation of partial miRNAs and the target genes during cold stress in the wild banana. **a**-**f**. The expression patterns of the 6 miRNAs were opposite to those of the related targets. **g**-**n**. The expressions of miR172 and the related targets by qPCR (**g**-**i**: different *sin3* gene members; **j**-**n**: *ada1*s). Duncan’s multiple range test: * *P* < 0.05; ** *P* < 0.01; the number of biological replicates = 3

The 11 miRNAs and the related targets were selected for validation by the real-time quantitative PCR (qPCR), and the results showed in Fig. 6. The expression patterns of the 6 target genes, including *sulfurylase* gene (target of mit-miR395h), *SOD* gene (target of mit-miR398b), *calcium-binding site* gene (target of novel_30), *SAM-methyltrans-ferase* gene (target of mit-miR172c-3p), *GRAS* gene (target of mit-miR171b), *SBP-box* gene (target of mit-miR156g), were opposite to those of the related miRNAs, which implied the 6 targets were negatively regulated by the 6 miRNAs (Fig. 6a-f).

Furthermore, the miR172 family analysis indicated that miR172 coordinately regulated many biological processes, including histone acetylation/deacetylation. During cold stress in the wild banana, 2 target candidate genes-histone acetylation activatoror ada1 targeted by miR172a, miR172b, miR172c, miR172e-3p, and miRNA172c-3p, and the deacetylation suppressor gene *SWI-independent-3* (*Sin3*) (*histone deacetylase interacting*, the first identified genetically as a global regulator of transcription) targeted by miRNA172b, miRNA172b, miRNA172c-3p, miR172e-3p, were predicted, implying that miR172 coordinately regulated both histone acetylation and histone deacetylation. Therefore, their qPCR assays were conducted, and the experimental results (Fig. 6g-n) showed that the miR172 members negatively regulated the targets *ada1* and *sin3*, and the highest expression levels of the 4 miRNAs targeting ada1 occurred at 13 °C and their target *ada1* also expressed at the lowest levels at the temperature of 13 °C but the expression levels of *ada1* at 4 °C and 0 °C were higher than that at 28 °C, which suggested that at the critical growth temperature of 13 °C, the histone acetylation levels were also at the lowest levels, resulting in the lowest gene expression levels, which led to the lowest metabolic activities, and further led to growth ceased; however, at the chilling temperature of 4 °C and the 0 °C treated temperature, the expression levels of *ada1* were higher, and the histone acetylation levels were higher, which resulted in higher expression levels of cold stress responsive genes for cold acclimation during cold stress in the wild banana. In the meantime, the highest expression levels of the 2 members of the target *sin3* occurred at 0 °C as the ada1 did, and the other member at 4 °C, which suggested that the different members might function at chilling or 0 °C treated response to the cold stress in the wild banana. However, the histone acetylation and histone deacetylation were of the opposite functions, and the results showed that during cold stress, the expression of some genes were up-regulated but the others were down-regulated, and they were all targeted by miR172 family, although the miR172 family members differed to some extents, and which suggested that miR172 should regulate both histone acetylation and histone deacetylation via targeting different target genes for both histone acetylation and histone deacetylation, and the gene up-regulation and down-regulation during cold stress were coordinately regulated by the same miRNA family even the same member of the miRNA family, which further indicated that the miRNA could synchronize different even opposite biological processes for cold acclimation in the wild banana.

### Changes of enzymatic and non-enzymatic antioxidant system during cold stress in the wild banana

The activities of SOD, POD and CAT, and contents of H_2_O_2_ were examined during cold stress in the wild banana. SOD, POD and CAT belonged to the enzymatic antioxidant system. The results showed that the activities of SOD, POD and CAT were the highest at 4 °Cand slightly decreased at 0 °C, but all of the treatments were higher than the 28 °C (CK), which indicated that the enzymatic antioxidant system was activated for cold acclimation during cold stress in the wild banana (Additional file 19). Besides, the highest content of H_2_O_2_ occurred at 4 °C and decreased lightly at 0 °C, but all of the treatments were higher than the 28 °C (CK) (Additional file 19), which indicated that the contents of H_2_O_2_ increased during cold stress in the wild banana. Asai S (2008) [107] reported that the H_2_O_2_ accumulation could promote NO production, which also enhanced the capacity of ROS scavenging through MAPK pathway.

## Discussion

### The cold responsive miRNAs in *Musa itinerans* and the comparative analysis among other different species

In our research, mapped to the Banana Genome A, there were 265 known miRNAs of 43 miRNA families, implying many miRNAs were involved in response to clod stress in the wild banana. Moreover, there were 41 novel miRNAs, and among which the numbers of miRNA* increased remarkably at all the cold stress temperatures compared with those of the control (28 °C), suggesting that the miRNA* should also likely play roles in response to cold stress in the wild banana. Besides, the species and numbers of the ta-siRNAs varied in different temperature treatments, especially the species peaked at 4 °C. The highest numbers were at 13 °C. All of them indicated that the ta-siRNAs were also involved in the response to cold stress in the wild banana.

Cluster analysis of DE miRNAs indicated that some miRNAs were up-regulated, whereas some were down-regulated in the all the cold stress treatments. However, some others were specifically up-regulated or down-regulated at one or two cold stress treatments, implying that these miRNAs were specific for environmental chilling or 0 °Ctreated temperature treatments in the wild banana. Interestingly, some miRNAs were significantly up-regulated or down-regulated at one cold-stress temperature (at 0 °C, 4 °C or 13 °C), which suggested that there existed different mechanisms for 0 °C treatment, chilling or growth retardation during cold stress in the wild banana.

Among all the DE miRNAs, most of them were reported to be cold-responsive [20, 21, 108], such as miR156, miR159, miR160, miR164, miR166, miR167, miR168, miR169, miR170, miR171, miR319, miR393, miR396, miR397, miR398, etc. However, some miRNAs were seldom reported to be cold-responsive but expressed differentially during cold stress in the wild banana, such as miR395 [109], miR408 [110], miR172 [111], etc. The findings suggested that those miRNAs maybe play key roles in response to cold stress for cold acclimation for the tropical plants such as banana.

Comparative profiles of miRNA expression among different species during cold stress revealed that some miRNAs such as miR397 and miR169 were similar with up-regulation in *Arabidopsis* [112], poplar [113] and *Brachypodium* [114] but some miRNAs such as miR168 and miR172 were different and among which miR168 was up-regulated in poplar and *Arabidopsis* but down-regulated in rice [115], and miR172 was up-regulated in *Arabidopsis* and *Brachypodium* but not in poplar, even more significantly differences in expression patterns between two cultivars in *Camellia sinensis* were found [116]. Compared with the cold responsive miRNAs in *Musa itinerans* with those of other species [109, 117, 118], miR397 and miR169 were also similar in up-regulation pattern during cold stress, and the miR408 was up-regulated at 4 °Cbut down-regulated in 0 °C. Interestingly, most of members of miR172 family showed the similarity with miR408 but a few members were all down-regulated during cold stress, which indicated that some miRNAs such as miR397 and miR169 shared the conserved expression profiles among different species during cold stress and some miRNAs such as miR408 and miR172 showed different expression profiles among different members of the miRNA family, or among different species, even among different cultivars of the same species, suggesting that they should be specific and diverse in functions.

### The target genes of DE miRNAs in response to cold stress in *Musa itinerans*

**Fig. 7:**
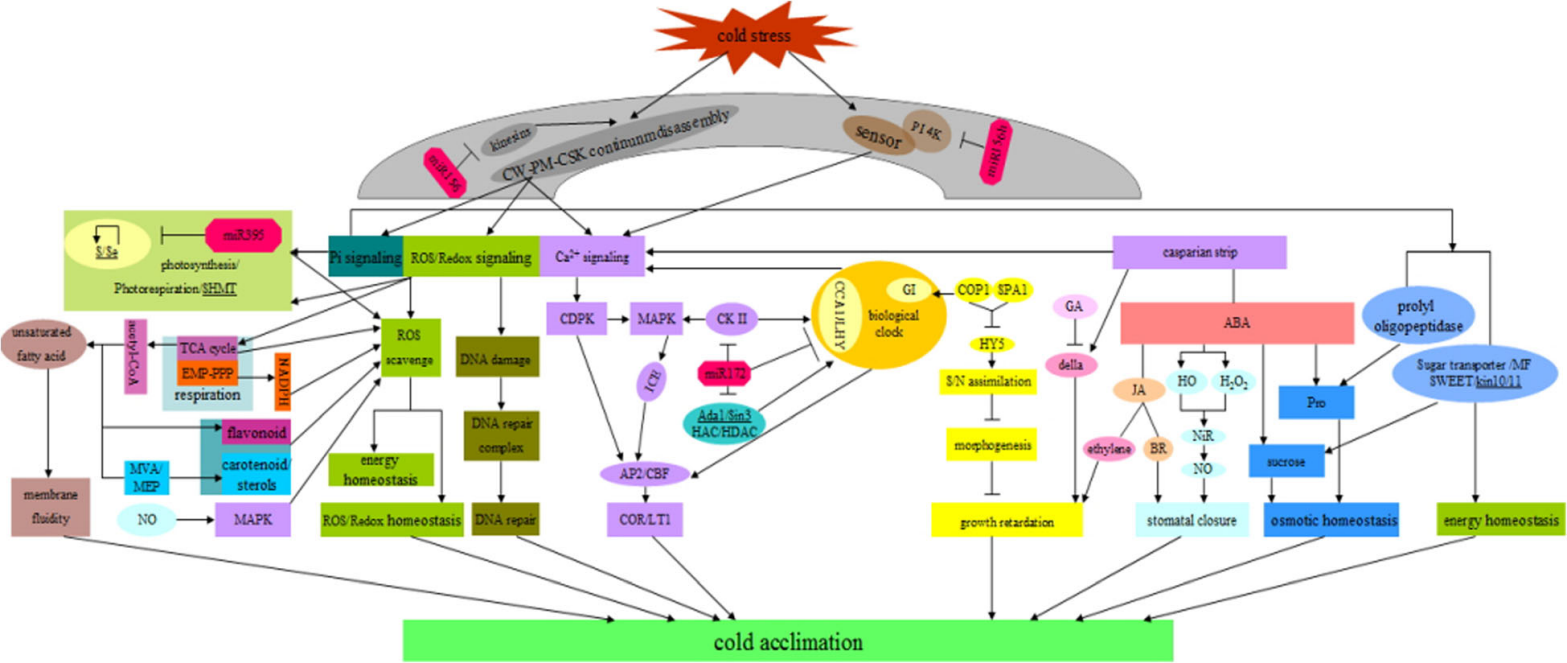
The diversified cold-responsive pathways of DE miRNAs targets during cold stress in the wild banana

The analyses of the targets candidates of DE miRNAs for cold acclimation during cold stress in *Musa itinerans* indicated that almost all of the pathways of cold responses reported before in plants existed during cold stress in the wild banana, suggesting the wild banana may be one of the model species, e.g., a tropical plant with a cold responsive mechanism. From the target genes of DE miRNA during cold stress, the outline of functions of the target genes could be inferred as follows: in the wild banana, during cold stress, both that the membrane sensor of the low temperature sensed cold signal and activated the phosphatidylinositol signaling system pathway, and that the membrane fluidity changed after cold stress resulted in disassembly of cell wall-plasma membrane-cytoskeleton continuum and further led to changes of cytoplasmic calcium and Pi levels and ROS-Redox status of photosynthesis and respiration, producing oxidative stress and ROS accumulation. The signals of these changes activated the down-stream low-temperature signaling transduction; in the meanwhile, the casparian strip, as a signal communication hub in response to hormones and propagation of calcium waves [119], also activated the down-stream low-temperature signaling transduction (Fig. 7).

### Sensing and transduction of cold signals during cold stress in the wild banana

In the wild banana, the cold signal was sensed and transduced by phosphatidylinositol signaling system pathway during cold stress, producing the messenger of cytoplasmic calcium waves, which was similar to that in human [105, 120] and *Arabidopsis* [106] and implied that there existed the similar cold sensing and transduction system in the wild banana. In the meantime, the membrane fluidity changed after cold stress resulted in disassembly of cell wall-plasma membrane-cytoskeleton continuum regulated by kinesins, which also produced the messenger of cytoplasmic calcium waves as well as a decrease of Pi levels and an increase of ROS-Redox status of photosynthesis and respiration, producing oxidative stress and ROS accumulation [121, 122]. Furthermore, calcium waves activated CDPK and MAPK cascades [123]. In plants, there are several known classes of Ca^2+^ binding sensory proteins, including calmodulins, CDPKs, and calcineurin B-like proteins [124]. CDPKs are implicated as major primary Ca^2+^ sensors and in response to environmental stress such as cold stress, and CDPK and MAPK signal transduction cascades become activated in parallel in plants [123]. Recent studies revealed that the casparian strip is not only a regulator of water and nutrient uptake, but also probably acts as signal center for hormone-mediated control of growth, and it may be considered as a signal communication hub in response to the external environment, facilitating the activation of hormone signaling pathways and the propagation of calcium waves [119, 125–127]. It is well known that an endodermis with casparian strip always occurs in roots, but it also occurs in stems and leaves of some vascular plants [128]. Yang et al. (2015) [118] reported that the casparian strip was related to the cold tolerance, and a casparian strip domain-like gene, *CASPL*, negatively altered growth and cold tolerance. In the wild banana, during cold stress, the *CASPL* gene for DE miRNA was predicted, so we inferred that *CASPL* might likely further activate the hormone-mediated responses to cold stress via signaling of hormones and propagation of calcium waves which derived from the phosphatidylinositol signaling system pathway.

### ROS scavenging and DNA repair for cold acclimation during cold stress in the wild banana

ROS comprises singlet oxygen and the reduced forms of oxygen, i.e., superoxide (O^2−^), hydrogen peroxide (H_2_O_2_) as well as the hydroxyl radical (OH^−^). During cold stress, respiration of mitochondria and photosynthesis of chloroplast produce oxidative stress and lead to ROS accumulation [129–131], and then the mechanism of ROS scavenging was activated and cleaved ROS for alleviating oxidative stress [129–132]. Oxidative stress with high levels of ROS causes damage to lipids, proteins and DNA [132]. In the wild banana during cold stress, the ROS-scavenging pathways are as follows: i) Enzymatic antioxidant system: the targets of DE miRNAs such as POD, PPO, SOD, CAT and APX, GPX, GR and GPOX were predicted. ii) Non-enzymatic antioxidant system, including GSH\ASH system, VitE\VitC system, secondary metabolites as antioxidants, i.e., flavonoids, steroids, carotenoids and anthocyanins, etc. The related synthetases or clastic enzymes of DE miRNAs were also predicted. The plant achieved energy homeostasis and ROS/Redox homeostasis through ROS scavenging to alleviating oxidative stress for cold acclimation during cold stress in the wild banana. Besides, in the wild banana during cold stress, the respiration of mitochondria and photosynthesis of chloroplast produced oxidative stress and led to ROS accumulation, which further caused DNA damage and then DNA repair. The genes related to DNA repair complex of DE miRNAs were predicted in the wild banana, which finished DNA repair for cold acclimation.

### The stomatal closure and growth retardant for cold acclimation during cold stress in the wild banana

In the wild banana, during cold stress, the respiration of mitochondria and photosynthesis of chloroplast produce oxidative stress and lead to ROS accumulation, including accumulation of H_2_O_2_ [133], and the H_2_O_2_ accumulation further promoted NO production by increase of NiR, finally NO promoted stomatal closure through the pathway of Ca^2+^/CAM or cGMP, which led to cold acclimation. Besides, NO also enhanced the capacity of ROS scavenging through MAPK pathway [107].

S and Se enhanced the H_2_O_2_ accumulation by sulfur assimilation, and Se induced sulfur starvation to enhance sulfur assimilation, which further led to stomatal closure by NO production for cold acclimation [76, 134–136]. Occurrence of the genes of DE miRNAs related to S and Se assimilation indicated that addition of S and Se might be helpful for cold acclimation during cold stress in the wild banana.

ABA is considered as a master regulator of responses to abiotic stresses [137–140]. In the wild banana, during cold stress, the biosynthesis of ABA pathway is activated, the related genes of DE miRNAs, such as *phytoene synthase* (*PSY*), *PDS* and *zeaxanthin epoxidase* (*ZEP*), were predicted. On one hand, the ABA signaling from the casparian strip enhances the production of H_2_O_2_ by NAD(P)H oxidases, and H_2_O_2_ further promotes NO production by up-regulation of NiR, finally NO promotes stomatal closure via the pathway of Ca^2+^/CAM or cGMP; on the other hand, ABA, as a plant growth retardant, retarded the plant growth for cold acclimation [141]. Furthermore, ABA also promoted NO production through the pathway of enhancement of HO (Heme oxygenase or haem oxygenase) activity to increase CO accumulation, and CO could rise the NO biosynthesis by the increase of NiR, and finally promoted stomatal closure for cold acclimation [142].

JA and BR also interacted with ABA to promote stomatal closure [137]. In the wild banana, during cold stress, the key enzymatic gene *SQLE* increased, which resulted in production of more steroids including BRs. In the meanwhile, the key enzymatic gene *FAD* also increased, which resulted in production of more unsaturated fatty acids, and further production of JA. Therefore, it is speculated that in the wild banana, JA and BR by means of the increase of *SQLE* and *FAD* levels interacted with ABA, and further promoted stomatal closure and retarded growth for cold acclimation during cold stress. Furthermore, the more steroids also promoted membrane fluidity.

Ethylene (ET) promoted senescence and retards plant growth [143]. Although GA promoted plant growth but DELLA inhibited the GA response to growth, and therefore it reduced the growth [144]. Besides, JA in concert with ET repressed cell cycle processes and expansion of leaf cells by suppressing AUXs [144–146]. In the wild banana, during cold stress, the key enzymatic genes of DE miRNAs in the pathway of ET biosynthesis, including *L-aspartate synthetase*, *L-methionine synthetase* and *S-adenosylmethionine synthetase* (*SAM*), etc., were predicted, suggesting ET accumulation. In the meantime, the *DELLA* gene of DE miRNAs was also predicted, which was negatively related to GA responses to growth [137]. Therefore, it was conferred that in the wild banana, ET biosynthesis produced more ET and the *DELLA* gene to suppress the GA responses, together retarded the plant growth for cold acclimation during cold stress in the wild banana.

### Activation of photorespiration, TCA and PPP pathway in the metabolic pathways of photosynthesis and respiration for cold acclimation during cold stress in the wild banana

In the wild banana, during cold stress, the signals relating to altered Pi levels leaded to activating the expression activities of related enzymes in the Calvin cycle in the chloroplast. In the meantime, the key gene *SHMT* of DE miRNAs were predicted, which implied that the photorespiration increased. The reaction catalyzed by SHMT linked to amino acid and nucleotide metabolism, and SHMT was a key enzyme in photorespiration, and played a critical role in controlling cell damage caused by abiotic stress, such as in rice [147] and *Arabidopsis* [148]. The increase of photorespiration led to activating the enzymatic antioxidant system for ROS scavenging. In rice and *Zoysia japonica* [149], during cold stress, while the photorespiration increased, the *SHMT* expression level rose for cold acclimation [147]. Therefore, it is inferred that the photorespiration increased to activate the enzymatic antioxidant system for cold acclimation during cold stress in the wild banana.

In the mitochondria, the genes of the TCA cycle-related emzymes of DE miRNAs were predicted, and shown to cause to a higher production of Acetyl-CoA and further affected fatty acid biosynthesis and degradation pathways, which then resulted in enhancing the biosynthesis of unsaturated fatty acids and steroids for improving membrane fluidity. The enhancement of TCA cycle also enhanced the capacity of ROS scavenging. The more production of Acetyl-CoA, contributed to the synthesis of the carotenoids via MVA/MEP pathway, the synthesis of the steroids, the synthesis of the flavonoids and the anthocyanins, accompanied more production of the antioxidants of the secondary metabolites such as carotenoids, steroids, flavonoids and anthocyanins, etc., which enhanced the capacity of ROS scavenging for cold acclimation.

In the cytoplasm, EMP-PPP pathway (PPP pathway related genes) was also predictively activated during cold stress, which would produced more NADPH for ROS scavenging for further cold acclimation.

### Keeping osmotic homeostasis for cold acclimation during cold stress in the wild banana

The decreased temperatures lead to an acute Pi-limitation of photosynthesis in *Arabidopsis* [150]. During cold stress, and Pi concentrations decreased and resulted in cytosolic phosphate (Pi) starvation [151]. The changes in Pi concentration to metabolism contribute to cold acclimation, and the Pi distribution shifts towards the cytoplasm at low temperatures [151, 152]. The signals relating to altered Pi levels led to activating the expression of enzymes in the sucrose synthesis pathway, as well as in the relative activities of enzymes in the Calvin cycle [152]. In addition to the sugar accumulation, the low temperatures induced the accumulations of some other biochemical components for cold acclimation, such as proline and other cryoprotectants [153–155].

In the wild banana, during cold stress, the related genes of Pi starvation targeted by DE miRNAs, the genes related sucrose and proline synthesis of DE miRNAs such as *sugar transporter*, *major facilitator superfamily domain*, *SWEET*, *VIN1*, *prolyl hydroxylases*, *KIN10/11*, and the genes involved in the fructose and mannose metabolism pathway, including the rate-limiting enzyme in glycolysis-phosphofructokinase, were predicted, suggesting a production of more sugars and proline to keep osmotic homeostasis for cold acclimation. Meanwhile, the proline synthesis was also activated by ABA signaling [156]. Moreover, sucrose starvation and energy deficit induced the expression of *KIN10/11* during cold stress [157–159], and in the wild banana, *KIN10/11* might regulate sucrose homeostasis and energy homeostasis for cold acclimation during cold stress for cold acclimation.

In a word, during cold stress, the Pi starvation, sucrose starvation and energy deficit, as well as ABA signaling, might activate the expression of the genes related to sugar and proline accumulation for further cold acclimation in the wild banana.

### Activation of the CBF pathway and regulation by *CBF* regulating loop associated with the circadian rhythm for cold acclimation during cold stress in the wild banana

It is well known that CBF (belonging AP2/ERF) pathway was activated by Ca^2+^ signaling pathway via CDPK and MAPK cascades for cold acclimation during cold stress in plants [160, 161]. From the prediction and functional analysis of the targets for the DE miRNAs, it was inferred that in the wild banana, cold stress, cold sensing and transduction produced the Ca^2+^ oscillation or waves, and the most essential signaling pathway was that activated CDPK and MAPK cascades as well as the further CBF pathways for cold acclimation. Furthermore, we found that there might exist a CBF regulating loop associated with the biological clock.

In *Arabidopsis*, CCA1, LHY and TOC1 have been suggested as core components of the central oscillator [82–84]. COP1 acted through ubiquitylation and targeted degradation of HY5 [89–91]. SPA1 associates with COP1 to promote COP1 activity and suppress photomorphogenesis [162]. In the meanwhile, COP1 also regulated temperature sensitivity by controlling the degradation of GI, and further to remotely regulated CCA1 by GI [93]. In the meantime, GI sensed the low-temperature signal and it was associated with sucrose sensing and permitted metabolic input into circadian timing [163, 164]. HY5 regulated N assimilation [165] and S assimilation [94, 95]. CK2 phosphoryla-tion of CCA1 was necessary for its circadian oscillator function in Arabidopsis [88] and it also acted as an essential component of the MAPK signal transduction pathway, which activated the MAPK cascades during cold stress. In the wild banana, during cold stress, the key protein kinases of CK2 and COP1 might regulate biological clock (circadian rhythm) to synchronize related signaling pathways and further activated various cold acclimation pathways; in the meanwhile, COP1/SPA1 complex negatively regulated HY5 to alleviate growth of plants for cold acclimation through regulation sulfur and N assimilation by HY5. The biological clock regulates many physiological and biochemical processes including calcium signaling in plants [166]. Therefore, it could be inferred that calcium signaling, CDPK, MAPK, CK2, CBF and the biological clock constituted a regulating loop, among them the biological clock might be the central regulator in the regulating loop during cold stress in the wild banana.

### MiR172 maybe played a central coordinating role especially by regulating *CK2* and the circadian rhythm in response to cold stress for cold acclimation in the wild banana

In the wild banana, there existed 10 members in miR172 family, which targeted as many as 1060 genes mapped to Banana Genome, among of which stu-miR172c-3p surprisingly targeted the most candidate genes (139 target candidates), such as the genes for *AP2/ARF domains*, including CBF; serine/threonine−/ dual-specificity protein kinase, including CK2; ada1/tada1, related to histone acetylation; histone deacetylase interacting (Paired amphipathic helix, Sin3), related to histone deacetylation; WD40/YVTN repeat-like-containing domain, related to ubiquitylation; kinesin, regulating assembly/disassembly of cytoskeleton-plasma membrane-cell wall continuums; S-adenosyl-L-methionine-dependent methyltransferase mida, related methylation; zinc finger (Ring/Fyve/ PHD-type), related to auxin signalling; Arp4, related to histone acetylation/chromatin remodeling; calmodulin binding protein-like, related to Ca^2+^ signaling; histone acetyltransferase ELP3, etc., which suggested that stu-miR172c-3p can simultaneously regulated phosphorylation of CK2, histone acetylation/deacetylation, histone methylation, ubiquitylation, chromatin remodeling, microtubule movement, assembly/disassembly of cytoskeleton-plasma membrane-cell wall continuums, sulfur metabolism, AP2/ARF transcriptional activities, etc., and which showed that miR172c-3p played roles in a wide range and coordinated many special key biological processes in responses to cold stress in the wild banana. Furthermore, the other members of miR172 family could also coordinately regulate many biological processes. In our experiment, it was validated by qPCR that miR172c-3p regulated both histone acetylation and histone deacetylation. It was reported that the LHY/CCA1 and CBF were regulated by histone acetylation and histone deacetylation [167–169]; besides, recently, Liu and Pile (2016) [170] reported that the miR172 target *Sin3* directly regulated genes involved in methionine catabolism and affects histone methylation, linking epigenetics and metabolism.

The analysis of target candidates of DE miRNAs in the wild banana indicated that CK2 and the biological clock were the core components of the responsive gene network during cold stress in the wild banana. CK2 acts as an essential component of the MAPK signal transduction pathway and the 4 members of *CK2* targeted by miR172c might activate the MAPK cascades in response to 0 °C treated temperature in the wild banana. In the meantime, *CK2*, together with *COP1* (targeted by miR172j) and *SPA1*, being also the targets of DE miRNAs in the circadian rhythm-plant pathway, were the key components of the biological clock. In plants, the biological clock regulates many physiological and biochemical processes such as the fluxes of solutes, ions such as K^+^, metabolic solutes such as sucrose, essential nutrients such as N and S, and signaling molecules calcium, etc. [97] and the rhythmic metabolism contributes to photosynthesis, starch metabolism, nutrient assimilation and redox homeostasis [98, 101]. Both CK2 and the key components of the biological clock were targeted by miR172, as did by many other essential cold-responsive biological processes. Therefore, miR172 (especially miR172c) maybe played an central coordinating roles, especially through regulating CK2 and the biological clock in response to cold stress for cold acclimation in the wild banana. It is reported that CK2 can be activated or inhibited by some chemicals such as polyamines, TBB, DMAT, NBC, etc. [171, 172], implying that the activators can be likely used as cold-resistant agents. The up-stream regulating mechanism of miR172 should be paid more attention to explore new clues to regulating miR172 for cold acclimation in the wild banana.

## Conclusions

In this study, the profiling of the cold-responsive miRNAs by RNA-seq was performed during cold stress in the wild banana (*Musa itinerans*). The 265 known mature miRNAs and 41 novel miRNAs were totally obtained. Cluster analysis of differentially expressed (DE) miRNAs indicated that some miRNAs were specific for chilling or 0 °C treated responses, and most of them were reported to be cold-responsive; however, some were seldom reported to be cold-responsive in response to cold stress, e.g., miR395, miR408, miR172, suggesting that they maybe play key roles in response to cold stress in the wild banana. The GO and KEGG pathway enrichment analysis of DE miRNAs targets indicated that there existed diversified cold-responsive pathways, and miR172 was found likely to play a central coordinating role in response to cold stress, especially in the regulation of *CK2* and the circadian rhythm. Finally, qPCR assays indicated the related targets were negatively regulated by the tested DE miRNAs during cold stress in the wild banana. The profiling of miRNAs by RNA-seq in response to cold stress in the plants of the wild banana (*Musa itinerans*) was reported for the first time, which provided insight into the roles of miRNAs during cold stress, and would be helpful for alleviating cold stress and cold-resistant breeding in bananas.

## Methods

### Plant materials and cold stress treatments, RNA isolation

The first leaves from the field Sanming wild banana plants, which grow well and uniformly, were selected for the test. And the in vitro cultured plants of Sanming wild banana were placed at various temperature conditions (28 °C, 20 °C, 13 °C, 4 °C, 0 °C, − 2 °C, − 4 °C and − 6 °C) under the fluorescent light of 2000 lx throughout 12/12 h/d light-dark cycle, with the relative humidity of 70–80% for 24 h. After temperature treatment for 24 h, the leaves from the field plants and the in vitro cultured plants of Sanming wild banana were used to survey the morphological changes of the leaves in all the temperature conditions tested. All the treatments were performed with 3 biological replicates. Each treatment contained 20 seedlings. The field Sanming wild banana plants was cultivated and preserved in the Wild Banana Germplasm Bank of Institute of Horticultural Biotechnology of Fujian Agriculture and Forestry University, which belong to Fujian provincial Sharing platform for germplasm resources of subtropical fruit trees and special economic crops.

The in-vitro plantlets after transplanting to the pots and cultivating for 1 month were used as the materials for treatments in the wild banana (*Musa itinerans*) from Sanming City of China, which were provided by the Institute of Horticultural Biotechnology, Fujian Agriculture and Forestry University, Fuzhou, Fujian Province, China. After transplanting for 1 month at 28 °C under the fluorescent light of 2000 lx throughout 12/12 h/d light-dark cycle, most of the seedlings were 15 to 18 cm in height and with 6 leaves. The seedlings with the uniform growth stage were selected for treatments in the experiment (Additional file 20). After sufficient watering for 2 days, the seedlings were put into the growth chambers at the growth temperature of 28 °C (as the control), and the low temperatures of 13 °C, 4 °C and 0 °C, respectively, under the fluorescent light of 2000 lx throughout a 12/12 h/d light-dark cycle (being synchronized with the natural light cycle) with the relative humidity of 70–80% for 24 h. After temperature treatment for 24 h, the first young leaf was detached from the top of each of the 10 seedlings at each temperature point (28 °C, 13 °C, 4 °C, 0 °C) for each biological replicate. All the treatments were with 3 biological replicates. For each replicate, the leaf samples of each 10 seedlings were cut into pieces with 0.5 cm in width in a direction perpendicular to the main vein and mixed well, and then they were randomly divided into the 3 sections, finally frozen in liquid N_2_ and stored at − 80 °C for further 3 different experiments of extract of the total RNA for RNA-sequencing and q-PCR assay and determination of the physiological changes, respectively.

The total RNA was extracted from the banana leaves according to the method by Liu et al. (2015) [10]. The total RNA integrity was qualified, and the RIN values of the samples from the treatments at 0 °C, 4 °C, 13 °C, 28 °C were between 7.7 and 8.1. The total RNA was used for further sequencing and qPCR analysis.

### Libraries preparation and sequencing

Four libraries were constructed and sequenced by Novogene (Beijing, China) using the Illumina HiSeq-4000 platform, and the details are referred to Bi et al. (2015) [49].

### Data analysis

Raw data were processed and the reads that did not conform the criteria were removed. The small RNA tags were mapped to reference sequence of the A genome of banana by Bowtie [172] and miRBase22.0. Modified software mirdeep2 [173] and sRNA-tools-cli [64] were used to analyse the miRNA structure. The miRNAcounts and the base bias both on the first position of the identified miRNAs with certain length and on each position of all the identified miRNAs. The novel miRNAs were predicted by online miREvo [174] and mirdeep2 [173], through exploring the secondary structure, the Dicer cleavage site and the minimum free energy of the small RNA tags. Known miRNA and novel miRNA precursor used miFam. dat (http://www.mirbase.org/ftp.shtml) and Rfam (http://rfam.sanger. ac.uk/search/) to analysis for miRNA families respectively. The target genes of the miRNAs were predicted by psRobot_tar in psRobot [175]. The DEGseq (2010) R package [65] was used to analysis DE miRNAs of four samples. *P*-value was adjusted using qvalue [176]. qvalue< 0.01 and |log2(foldchange)| > 1 was set as the threshold for significantly DE by default. The target genes candidates of DE miRNAs were used to GO and KEGG enrichment analysis [177–179]. GOseq based wallenius non-central hyper-geometric distribution [180] and KOBAS [181] software were implemented for GO enrichment analysis and assessing the statistical enrichment of the target gene candidates in KEGG pathways.

### Real-time quantitative PCR validation of differentially expressed miRNAs and novel miRNAs and their target genes

The total RNAs extracted from the leaves after cold treatments (including the control) were further used for reverse transcript of miRNA and the target mRNA. All the treatments were with 3 biological replicates, and each replicate was pooled from 10 seedlings. 1 μg of DNase I-treated total RNA was used in a reverse transcription reaction with the TransScript® miRNA first-strand cDNA synthesis supermix (TransGen, Beijing, China) and the Prime-Script® RT reagent Kit (Takara, Dalian, China). *U6* and *CAC* were respectively used as the internal control for the miRNAs and their targets genes. The expression validation of miRNAs and their targets genes were performed by LightCycler 480 (Roche). The reaction systerm and procedures were referred to Liu et al. (2015) [10]. The expression levels of the miRNAs and their target genes were quantified using the comparative 2^-ΔΔ^Ct method [182]. The primer sequences were designed using Primer 3 input soft-ware and are listed in Additional file 21. SPSS (Statistical Product and Service Solutions) test was used to assess statistically significant differences in the data of the results in this experiment.

### Determination of the acvivities of enzymatic antioxidant system and the contents of H_2_O_2_ during cold stress in the wild banana

The acitivities of SOD, POD, CAT and the contents of H_2_O_2_ were determined by spectrophotometer method. The kits were bought from Suzhou Comin Biotechnology Co. Ltd.

We ensure that we have complied with the Convention on the Trade in Endangered Species of Wild Fauna and Flora during all the processes of sampling of experimental research on plants (either cultivated or wild banana).

## Additional files

Additional file 1: **Figure S1.** The lowest temperature of Sanming city for the recent 5 years. (PDF 40 kb)

Additional file 2: **Table S1.** The results of sRNA sequences from four wild banana libraries mapped to genome A. (XLS 21 kb)

Additional file 3: **Table S2.** Numbers of reads for each small RNA classification identified, the map of known, novel miRNA. (XLS 16 kb)

Additional file 4: **Table S3.** The length distribution of sequencing reads from the 4 small RNA libraries. (XLS 24 kb)

Additional file 5: **Table S4.** Detailed information of the known miRNAs mapped to Banana Genome A. (XLS 156 kb)

Additional file 6: **Table S5.** Detailed information of identified novel miRNAs mapped to Banana Genome A. (XLS 56 kb)

Additional file 7: **Table S6.** ta-siRNA mapped to Banana Genome A. (XLS 42 kb)

Additional file 8: **Figure S2.** Heatmap of DE miRNAs during cold stress in the wild banana. (PDF 137 kb)

Additional file 9: **Figure S3.** Venn diagrams showing the number of DE miRNAs in two or three groups. (PDF 77 kb)

Additional file 10: **Table S7.** Annotation of miRNA targets mapped to Banana Genome A. (XLS 499 kb)

Additional file 11: **Table S8.** Numbers of miRNA targets. (XLS 42 kb)

Additional file 12: **Figure S4.** GO enrichment analysis mapped to Banana Genome A during cold stress in the wild banana. (PDF 802 kb)

Additional file 13: **Table S9.** Significant GO terms for target genes of differentially miRNAs mapped to Banana Genome A. (XLS 5758 kb)

Additional file 14: **Table S10.** KEGG enrichment analysis mapped to Banana Genome A. (XLS 239 kb)

Additional file 15: **Table S11.** The top 20 enriched pathways identified for target genes mapped to Banana Genome A. (XLS 33 kb)

Additional file 16: **Figure S5.** The scatter diagrams of top 20 KEGG pathways enriched of target genes for DE miRNAs. (PDF 441 kb)

Additional file 17: **Table S12.** The frequencies of TF and non-TF targets. (XLS 22 kb)

Additional file 18: **Figure S6.** The targets for DE miRNAs enriched in the top 20 KEGG pathway. (PDF 1769 kb)

Additional file 19: **Figure S7.** The SOD, POD, CAT activities and H_2_O_2_ contents in the wild banana during cold stress. (PDF 1497 kb)

Additional file 20: **Figure S8.** The seedlings used from treatments in the wild banana from Sanming City. (PDF 139 kb)

Additional file 21: **Table S13.** The primers used in this study. (XLS 27 kb)

## Abbreviations

Ada1: Adaptor 1/Tada1: Transcriptional adaptor 1
AP2: APETALA2 transcription factors
APS: Adenosine-5′-phosphosulfate
ARF: Auxin response factor
BR: Brassinosteroids
Bzip: Basic region/leucine zipper motif transcription factors
CrtL-b: Carotene lycopene β-cyclase
DELLA: Aspartic acid–glutamic acid–leucine–leucine–alanine
DUF: Domain of unknown function
ERG1: Ergosterol biosynthesis protein 1
ET: Ethylene
FAD: Fatty acid desaturase
GA: Gibberellin
GGPP: Geranylgeranyl pyrophosphate
GRAS: Gibberelic Acid Insensitive Repressor of ga1–3 Scarecrow, GAI, RGA and SCR
JA: Jasmonic acid;
LUT1: Lutein 1
NAM: No apical meristem
PAPS: 3′-phosphoadenosine-5′-phosphosulfate
PDS: Phytoene desaturase
PSY: Phytoene synthase
q-PCR: Real-time quantitative PCR
SAM: S-adenosylmethionine synthetase
SBP-box: Quamosa promoter binding protein box
Sin3: SWI-independent-3
SIR: Sulphite reductase
SLIM1: Sulfur limitation 1
SQLE: Squalene epoxidase
TPRs: Tetratricopeptide repeat motif proteins
Tum1: Thiosulphate sulfurtransferase
WD40: WD repeat-containing protein, WD40 repeat is a short structural motif of approximately 40 amino acids
Zinc finger: Zinc-binding motif transcription factors
Z-ISO: 15-cis-zeta-carotene isomerase
