# Supplementary figures and images for: High-throughput sequencing of small RNAs revealed the diversified cold-responsive pathways during cold stress in the wild banana (Musa itinerans)
Source: BMC Plant Biol. 2018 Nov 29;18:308. doi: 10.1186/s12870-018-1483-2 (PMC6263057; doi:10.1186/s12870-018-1483-2)

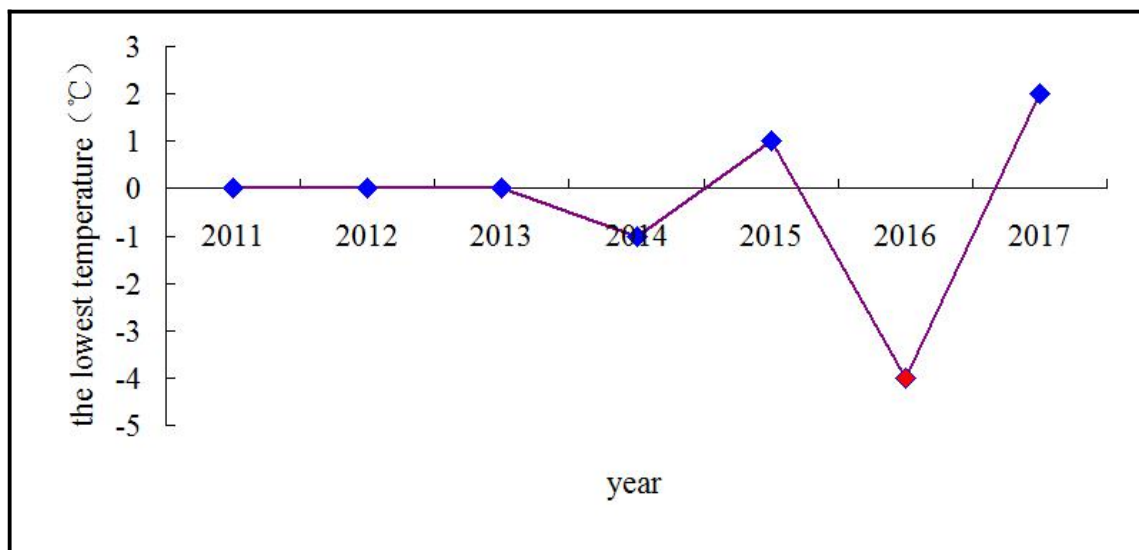

**Additional file 1 Figure S1 The lowest temperature of Sanming city for the recent 5 years.**

Supplement: Supplementary file 1 — Figure S1. The lowest temperature of Sanming city for the recent 5 years. (PDF 40 kb) [file 12870_2018_1483_MOESM1_ESM.pdf]

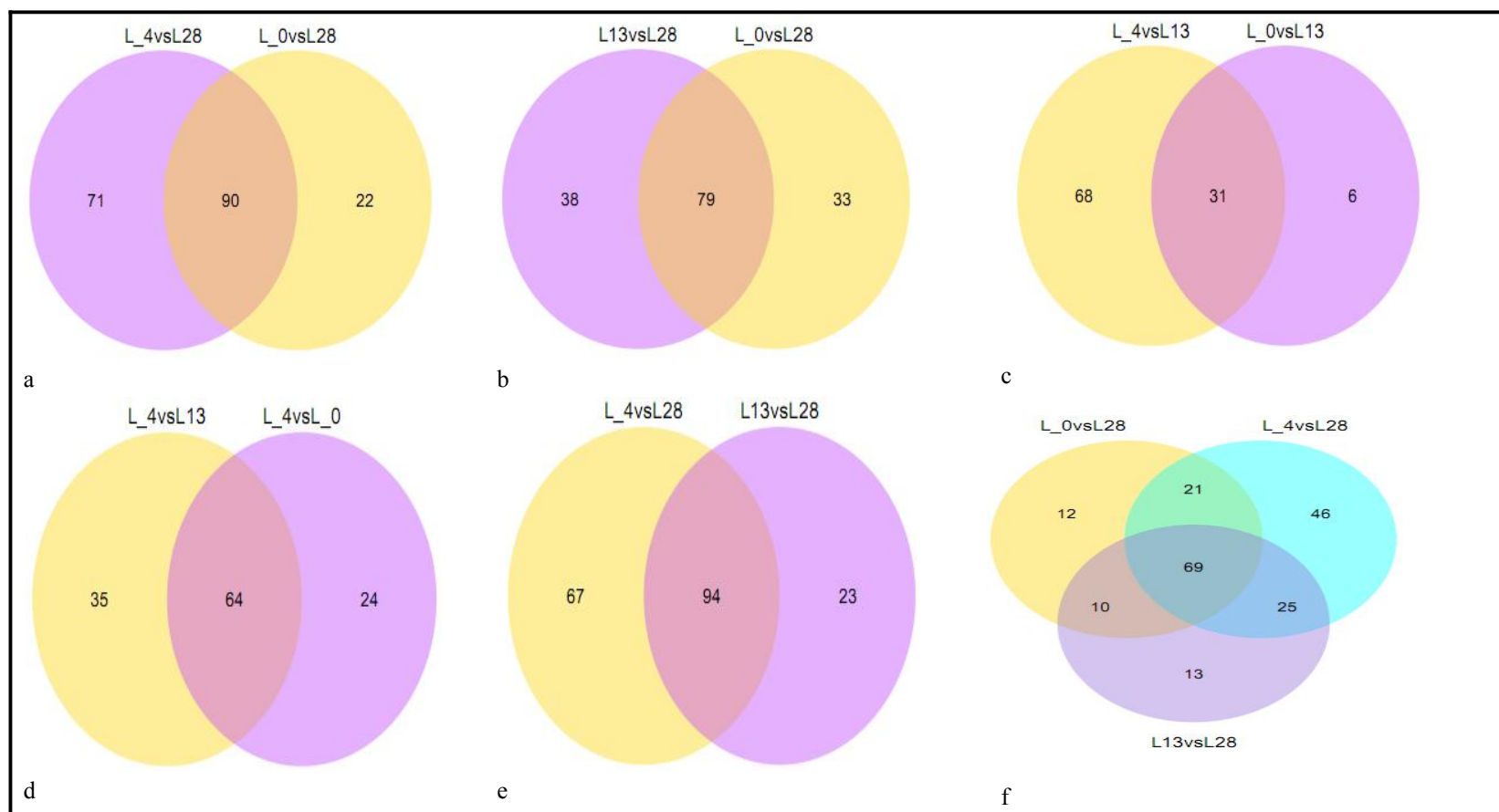

**Additional file 9 Figure S3 Venn diagrams showing the number of DE miRNAs in two or three groups.**

Supplement: Supplementary file 9 — Figure S3. Venn diagrams showing the number of DE miRNAs in two or three groups. (PDF 77 kb) [file 12870_2018_1483_MOESM9_ESM.pdf]
