# Supplementary material for: High-throughput sequencing of small RNAs revealed the diversified cold-responsive pathways during cold stress in the wild banana (Musa itinerans)
Source: BMC Plant Biol. 2018 Nov 29;18:308. doi: 10.1186/s12870-018-1483-2 (PMC6263057; doi:10.1186/s12870-018-1483-2)
Supplement: Supplementary file 8 — Figure S2. Heatmap of DE miRNAs during cold stress in the wild banana. (PDF 137 kb) [file 12870_2018_1483_MOESM8_ESM.pdf]

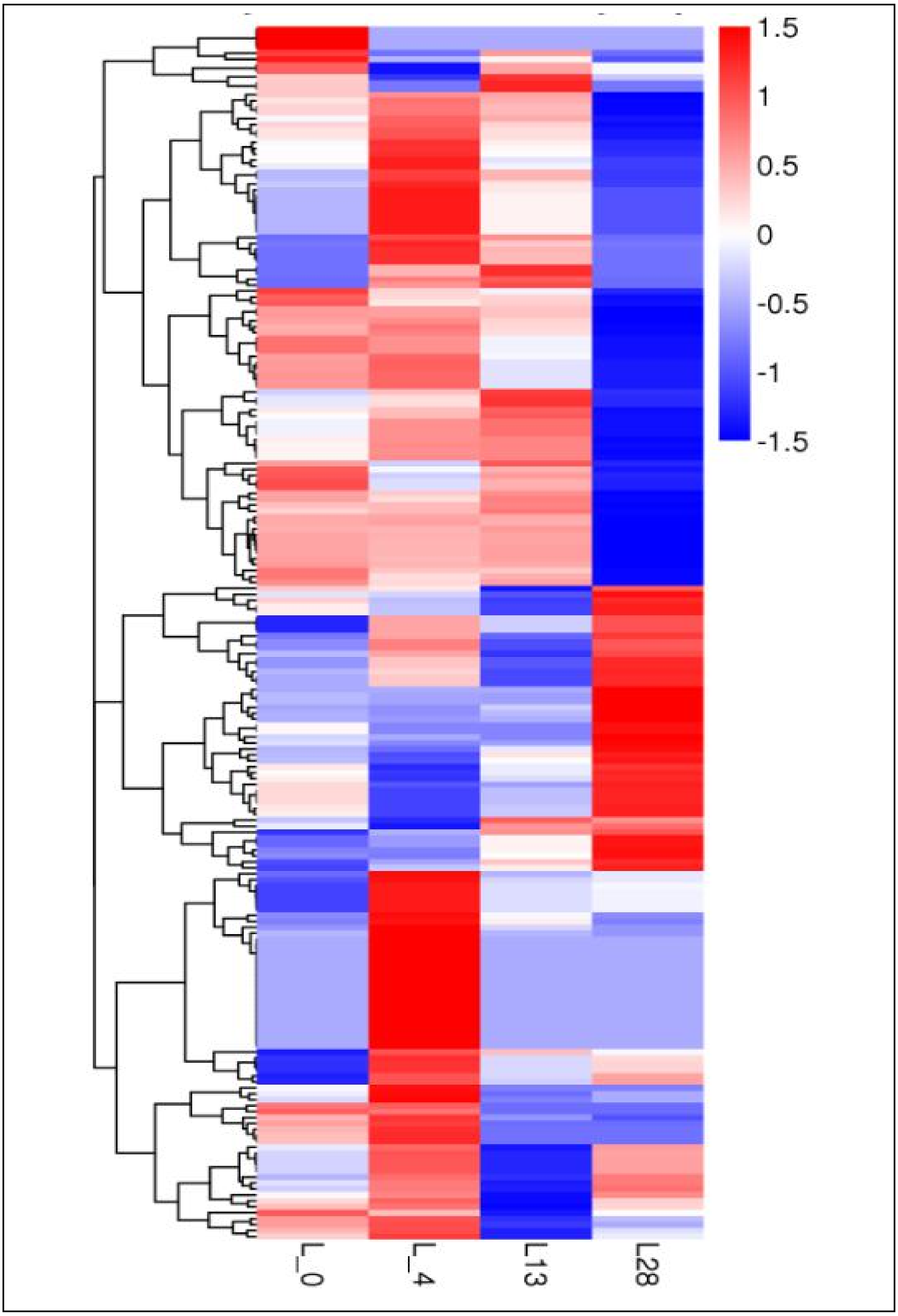


**Additional file 8 Figure S2 Heatmap of DE miRNAs during cold stress in the wild banana.** Red corresponds to up-expressed sRNAs; blue corresponds to down-expressed sRNAs. From red to blue corresponds to the numerical value of log10 (TPM+1) from big to small. L0, L4, L13, and L28 corresponds to 0℃, 4℃, 13℃and 28℃ library respectively.
