# Supplementary material for: High-throughput sequencing of small RNAs revealed the diversified cold-responsive pathways during cold stress in the wild banana (Musa itinerans)
Source: BMC Plant Biol. 2018 Nov 29;18:308. doi: 10.1186/s12870-018-1483-2 (PMC6263057; doi:10.1186/s12870-018-1483-2)
Supplement: Supplementary file 12 — Figure S4. GO enrichment analysis mapped to Banana Genome A during cold stress in the wild banana. (PDF 802 kb) [file 12870_2018_1483_MOESM12_ESM.pdf]

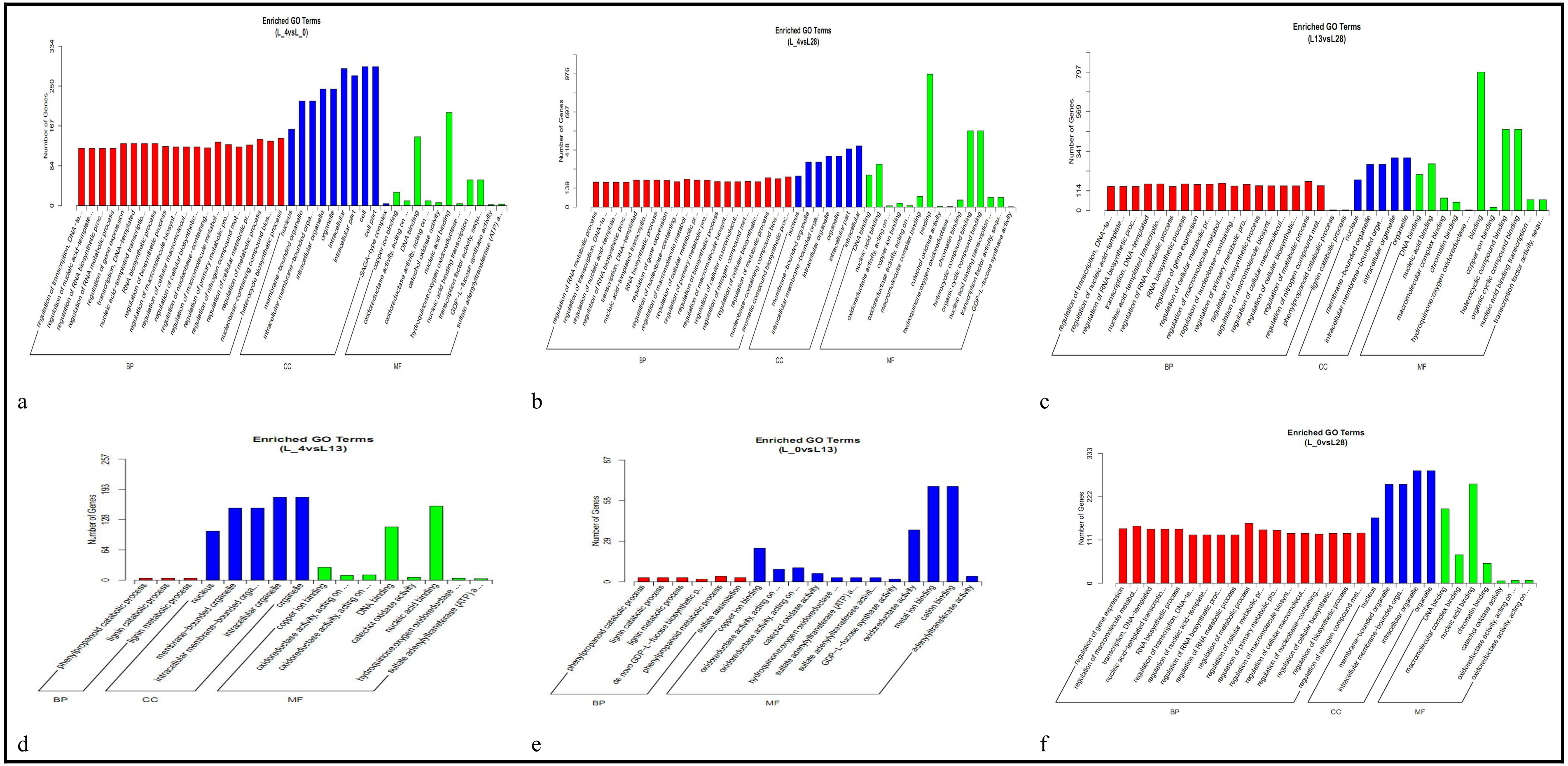

Additional file 12 Figure S4 **GO enrichment analysis mapped to Banana Genome A during cold stress in the wild banana.**
