# Supplementary material for: High-throughput sequencing of small RNAs revealed the diversified cold-responsive pathways during cold stress in the wild banana (Musa itinerans)
Source: BMC Plant Biol. 2018 Nov 29;18:308. doi: 10.1186/s12870-018-1483-2 (PMC6263057; doi:10.1186/s12870-018-1483-2)
Supplement: Supplementary file 16 — Figure S5. The scatter diagrams of top 20 KEGG pathways enriched of target genes for DE miRNAs. (PDF 441 kb) [file 12870_2018_1483_MOESM16_ESM.pdf]

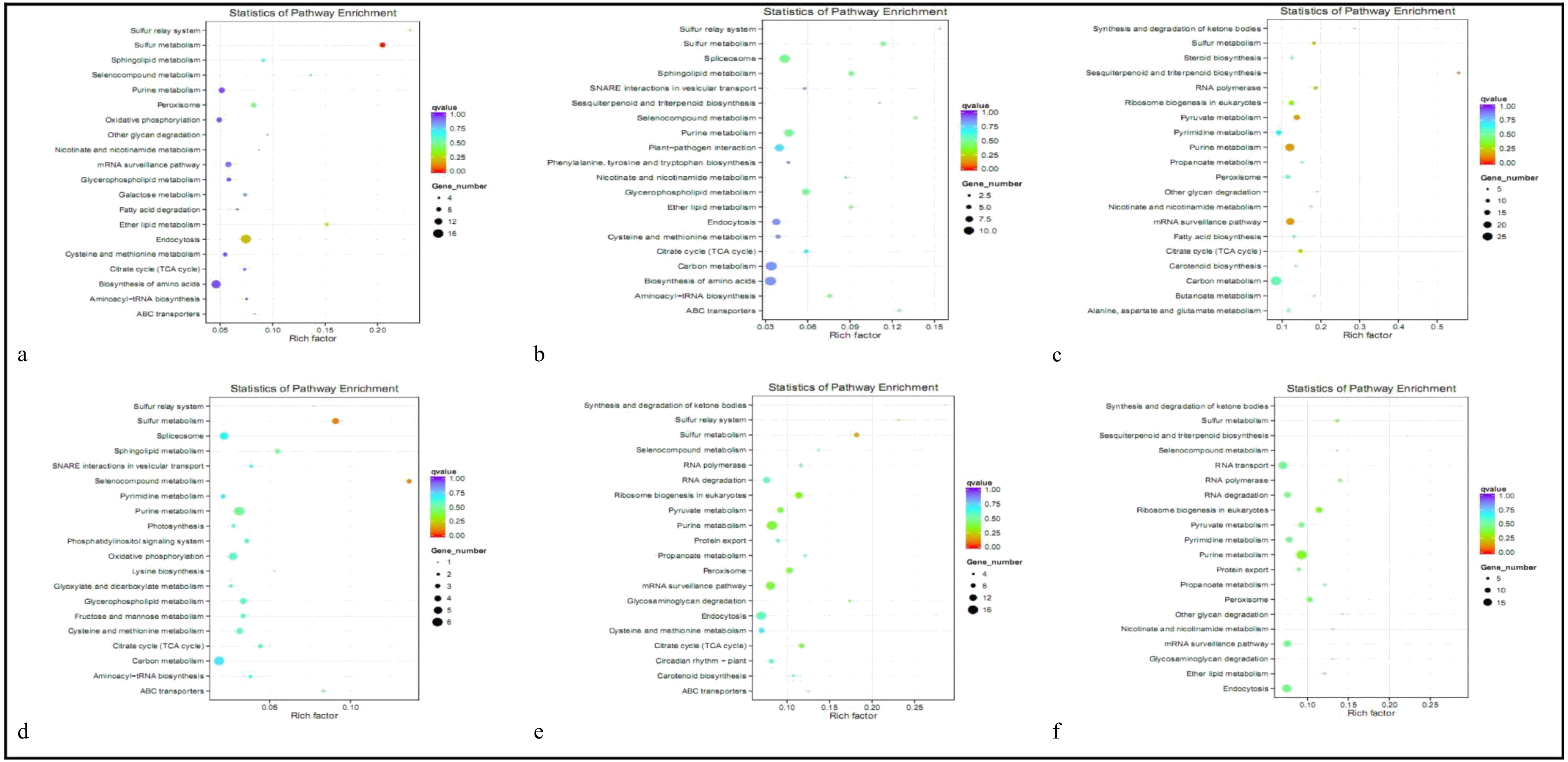

Additional file 16 Figure S5 The scatter diagrams of top 20 KEGG pathways enriched of target genes for DE miRNAs. A: L4 *vs* L0; B: L4 *vs* L13; C: L4 *vs* L28; D: L0 *vs* L13; E: L0 *vs* L28; F: L13 *vs* L28.
