# Supplementary material for: High-throughput sequencing of small RNAs revealed the diversified cold-responsive pathways during cold stress in the wild banana (Musa itinerans)
Source: BMC Plant Biol. 2018 Nov 29;18:308. doi: 10.1186/s12870-018-1483-2 (PMC6263057; doi:10.1186/s12870-018-1483-2)
Supplement: Supplementary file 18 — Figure S6. The targets for DE miRNAs enriched in the top 20 KEGG pathway. (PDF 1769 kb) [file 12870_2018_1483_MOESM18_ESM.pdf]

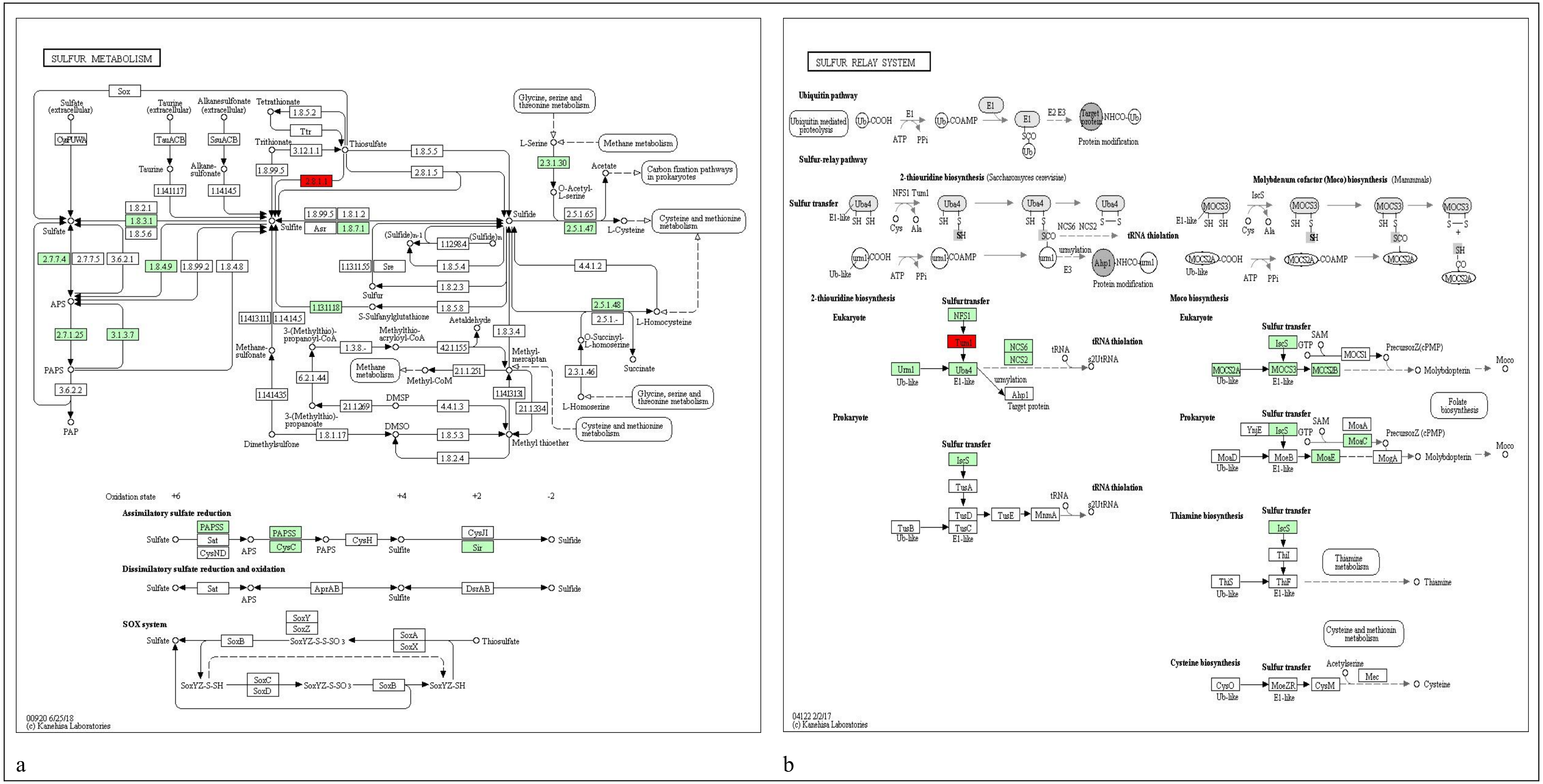
SULFUR METABOLISM
Oxidation state
Assimilatory sulfate reduction
Dissimilatory sulfate reduction and oxidation
SOX system
00920 6/25/18
(c) Kanehisa Laboratories
a
SULFUR RELAY SYSTEM
Ubiquitin pathway
Sulfur-relay pathway
Sulfur transfer
2-thiouridine biosynthesis
Molybdenum cofactor (Moco) biosynthesis
Moco biosynthesis
Eukaryote
Prokaryote
Thiamine biosynthesis
Cysteine biosynthesis
tRNA thiolation
04122 2/2/17
(c) Kanehisa Laboratories
b

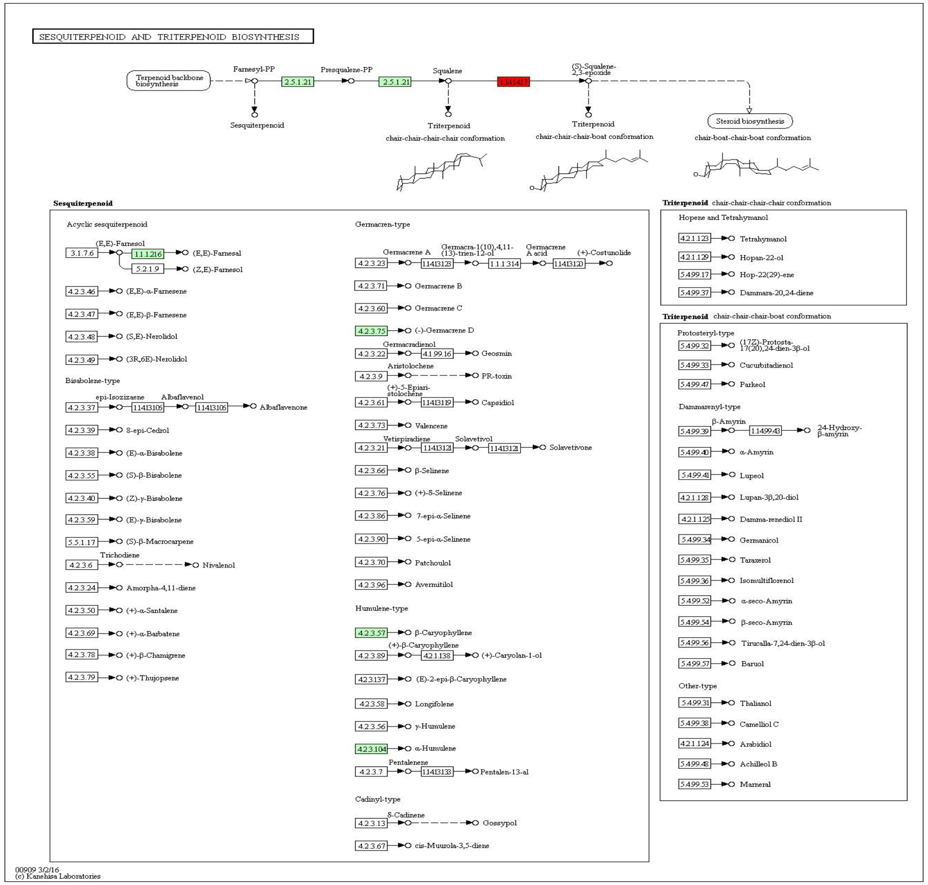
SESQUITERPENOID AND TRITERPENOID BIOSYNTHESIS
Terpenoid backbone biosynthesis
Farnesyl-PP
Presqualene-PP
Squalene
(S)-Squalene-2,3-epoxide
Sesquiterpenoid
Triterpenoid
chair-chair-chair-chair conformation
chair-chair-chair-boat conformation
Steroid biosynthesis
chair-boat-chair-boat conformation
Sesquiterpenoid
Acyclic sesquiterpenoid
Bisabolene-type
Germacren-type
Humulene-type
Cadinyl-type
Triterpenoid chair-chair-chair-chair conformation
Hopene and Tetrahymanol
Triterpenoid chair-chair-chair-boat conformation
Protosteryl-type
Dammarenyl-type
Other-type
00909 3/2/16
(c) Kanehisa Laboratories

c

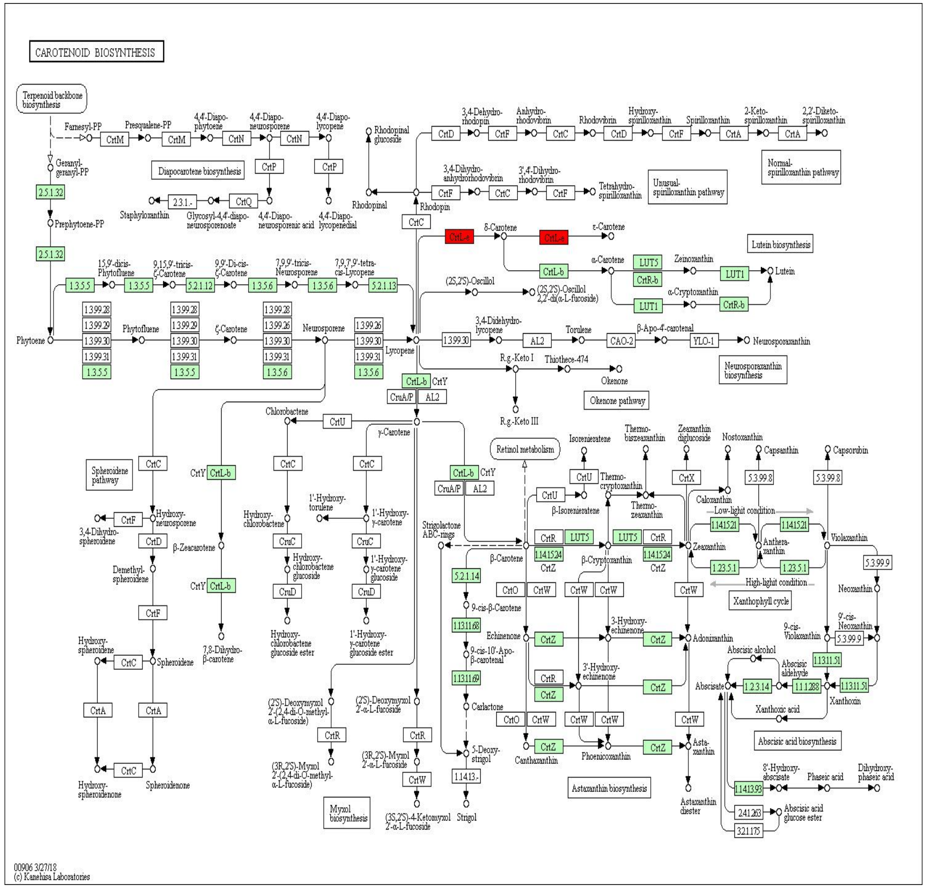
CAROTENOID BIOSYNTHESIS
Terpenoid backbone biosynthesis
Diapocarotene biosynthesis
Phytoene
Lycopene
Neurosporene
Lutein biosynthesis
Lutein
Zeaxanthin
Violaxanthin
Neoxanthin
Xanthoxin
Abscisate
Abscisic acid biosynthesis
Astaxanthin biosynthesis
Retinol metabolism
Okenone pathway
Neurosporaxanthin biosynthesis
Spheroidene pathway
Myxol biosynthesis
Xanthophyll cycle
00906 3/27/18
(c) Kanehisa Laboratories

d

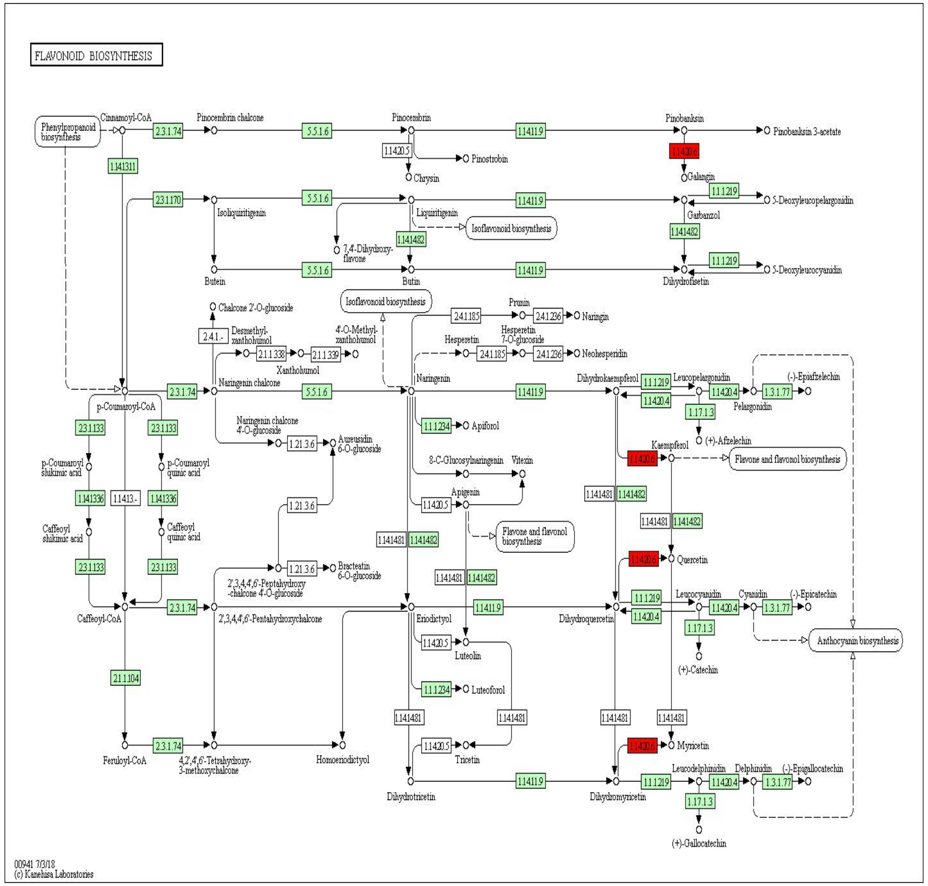
FLAVONOID BIOSYNTHESIS
Phenylpropanoid biosynthesis
Cinnamoyl-CoA
Pinocembrin chalcone
Pinocembrin
Pinobanksin
Pinobanksin 3-acetate
Pinostrobin
Chrysin
Galangin
Isoliquiritigenin
Liquiritigenin
Isoflavonoid biosynthesis
7,4'-Dihydroxy-flavone
Garbanzol
5-Deoxyleucopelargonidin
Butein
Butin
Dihydrofisetin
5-Deoxyleucocyanidin
Chalcone 2'-O-glucoside
Desmethyl-xanthohumol
4'-O-Methyl-xanthohumol
Xanthohumol
Prunin
Naringin
Hesperetin
Hesperetin 7-O-glucoside
Neohesperidin
p-Coumaroyl-CoA
Naringenin chalcone
Naringenin
Dihydrokaempferol
Leucopelargonidin
Pelargonidin
(-)-Epiafzelechin
(+)-Afzelechin
Naringenin chalcone 4'-O-glucoside
Aureusidin 6-O-glucoside
Apiforol
Kaempferol
Flavone and flavonol biosynthesis
p-Coumaroyl shikimic acid
p-Coumaroyl quinic acid
8-C-Glucosylnaringenin
Vitexin
Apigenin
Caffeoyl shikimic acid
Caffeoyl quinic acid
Quercetin
Bracteatin 6-O-glucoside
2',3,4,4',6'-Peptahydroxy-chalcone 4'-O-glucoside
Caffeoyl-CoA
2',3,4,4',6'-Pentahydroxychalcone
Eriodictyol
Dihydroquercetin
Leucocyanidin
Cyanidin
(-)-Epicatechin
Anthocyanin biosynthesis
Luteolin
(+)-Catechin
Luteoforol
Feruloyl-CoA
4,2',4',6'-Tetrahydroxy-3-methoxychalcone
Homoeriodictyol
Tricetin
Myricetin
Dihydrotricetin
Dihydromyricetin
Leucodelphinidin
Delphinidin
(-)-Epigallocatechin
(+)-Gallocatechin
00941 7/3/18
(c) Kanehisa Laboratories

e

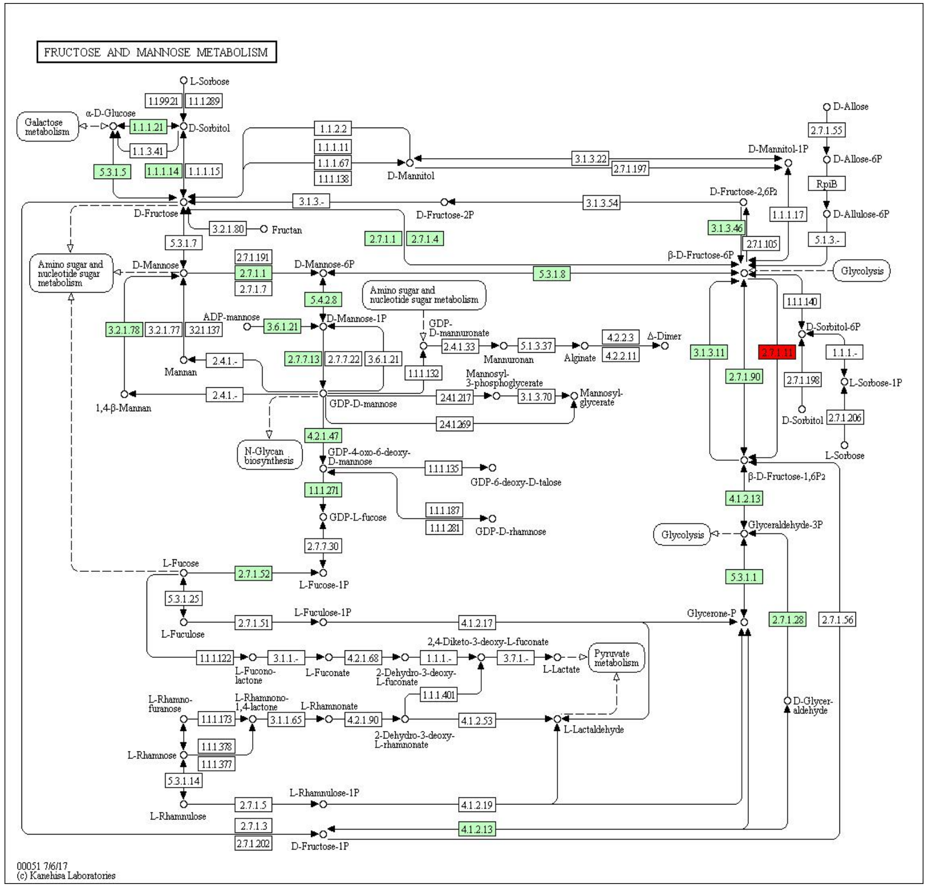
FRUCTOSE AND MANNOSE METABOLISM
L-Sorbose
Galactose metabolism
α-D-Glucose
D-Sorbitol
D-Mannitol
D-Mannitol-1P
D-Allose
D-Allose-6P
D-Allulose-6P
D-Fructose
D-Fructose-2P
D-Fructose-2,6P2
Fructan
Amino sugar and nucleotide sugar metabolism
D-Mannose
D-Mannose-6P
β-D-Fructose-6P
Glycolysis
ADP-mannose
D-Mannose-1P
GDP-D-mannuronate
Mannuronan
Alginate
Δ-Dimer
D-Sorbitol-6P
L-Sorbose-1P
D-Sorbitol
L-Sorbose
Mannan
1,4-β-Mannan
GDP-D-mannose
Mannosyl-3-phosphoglycerate
Mannosyl-glycerate
N-Glycan biosynthesis
GDP-4-oxo-6-deoxy-D-mannose
GDP-6-deoxy-D-talose
GDP-L-fucose
GDP-D-rhamnose
β-D-Fructose-1,6P2
Glyceraldehyde-3P
L-Fucose
L-Fucose-1P
L-Fuculose
L-Fuculose-1P
Glycerone-P
2,4-Diketo-3-deoxy-L-fuconate
L-Fucono-lactone
L-Fuconate
2-Dehydro-3-deoxy-L-fuconate
L-Lactate
Pyruvate metabolism
L-Rhamno-furanose
L-Rhamno-1,4-lactone
L-Rhamnonate
2-Dehydro-3-deoxy-L-rhamnonate
L-Lactaldehyde
D-Glyceraldehyde
L-Rhamnose
L-Rhamnulose
L-Rhamnulose-1P
D-Fructose-1P
00051 7/6/17
(c) Kanehisa Laboratories

f

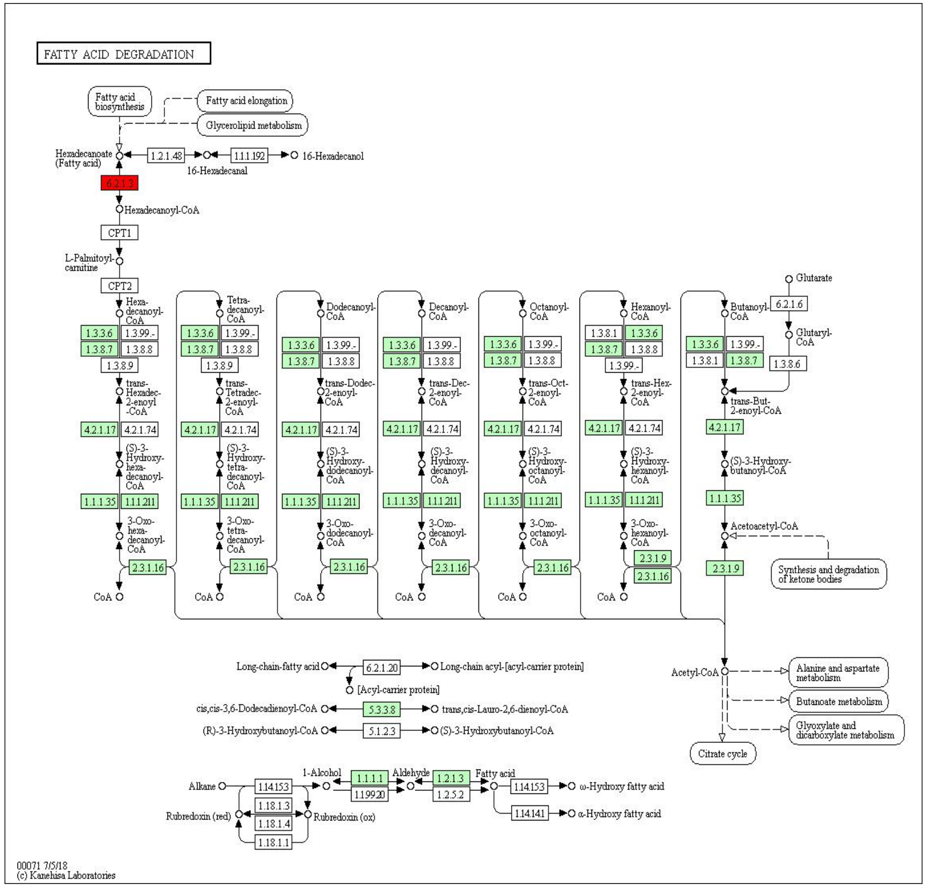
FATTY ACID DEGRADATION
Fatty acid biosynthesis
Fatty acid elongation
Glycerolipid metabolism
Hexadecanoate (Fatty acid)
16-Hexadecanal
16-Hexadecanol
Hexadecanoyl-CoA
CPT1
L-Palmitoyl-carnitine
CPT2
Glutarate
Glutaryl-CoA
Butanoyl-CoA
(S)-3-Hydroxy-butanoyl-CoA
Acetoacetyl-CoA
Synthesis and degradation of ketone bodies
CoA
Acetyl-CoA
Long-chain-fatty acid
Long-chain acyl-[acyl-carrier protein]
[Acyl-carrier protein]
cis,cis-3,6-Dodecadienoyl-CoA
trans,cis-Lauro-2,6-dienoyl-CoA
(R)-3-Hydroxybutanoyl-CoA
(S)-3-Hydroxybutanoyl-CoA
Alanine and aspartate metabolism
Butanoate metabolism
Glyoxylate and dicarboxylate metabolism
Citrate cycle
Alkane
1-Alcohol
Aldehyde
Fatty acid
ω-Hydroxy fatty acid
α-Hydroxy fatty acid
Rubredoxin (red)
Rubredoxin (ox)
00071 7/5/18
(c) Kanehisa Laboratories

g

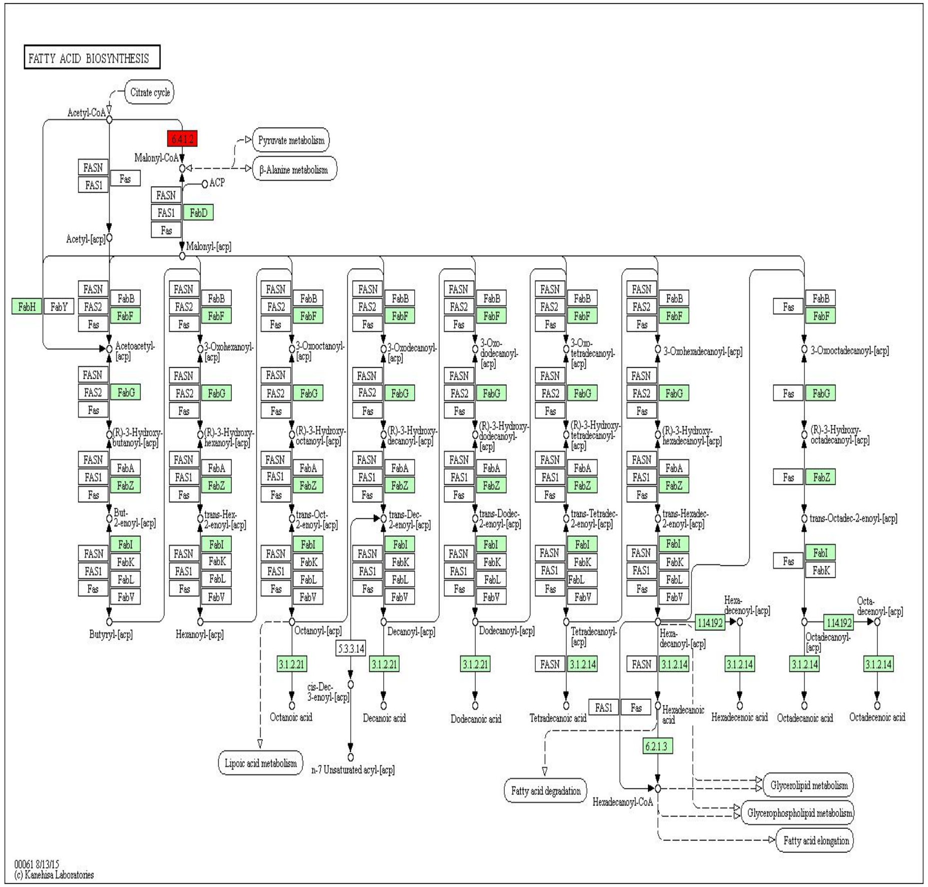
FATTY ACID BIOSYNTHESIS
Citrate cycle
Acetyl-CoA
Malonyl-CoA
Pyruvate metabolism
β-Alanine metabolism
ACP
Acetyl-[acp]
Malonyl-[acp]
Butyryl-[acp]
Hexanoyl-[acp]
Octanoyl-[acp]
Decanoyl-[acp]
Dodecanoyl-[acp]
Tetradecanoyl-[acp]
Hexadecanoyl-[acp]
Octadecanoyl-[acp]
Octanoic acid
Decanoic acid
Dodecanoic acid
Tetradecanoic acid
Hexadecanoic acid
Hexadecenoic acid
Octadecanoic acid
Octadecenoic acid
Lipoic acid metabolism
n-7 Unsaturated acyl-[acp]
Fatty acid degradation
Hexadecanoyl-CoA
Glycerolipid metabolism
Glycerophospholipid metabolism
Fatty acid elongation
00061 8/13/15
(c) Kanehisa Laboratories

h

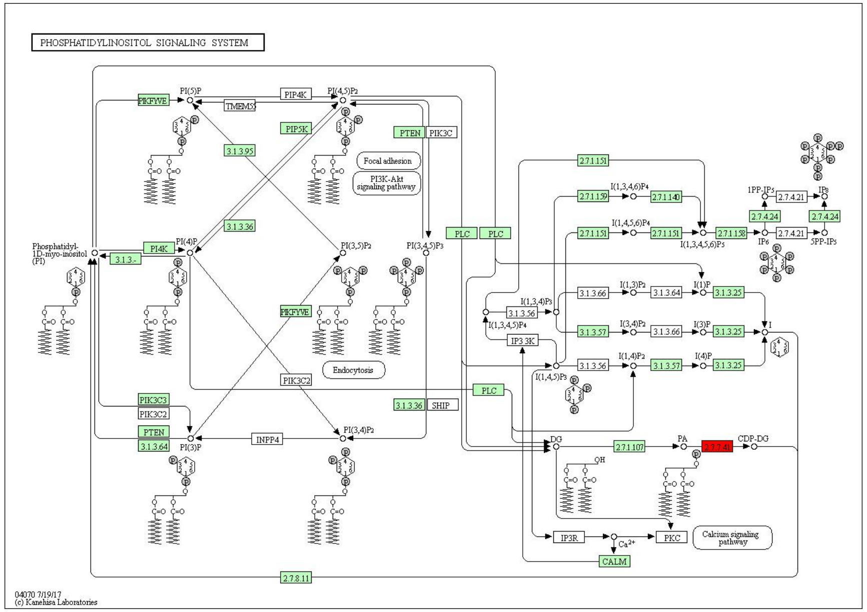

i

Additional file 18 Figure S6 The targets for DE miRNAs enriched in the top 20 KEGG pathway. a: sulfur metabolism in L4 *vs* L0, and the other five groups also in the top 20 pathway; b: sulfur relay system in L4 *vs* L0, the L4 *vs* L13, L0 *vs* L13 and L0 *vs* L28 also in the top 20 pathway; c: sesquiterpenoid and triterpenoid biosynthesis in L4 *vs* L28, the L4 *vs* L13 and L13 *vs* L28 also in the top 20 pathway; d: carotenoid biosynthesis in L4 *vs* L28, and the L0 *vs* L28 also in the top 20 pathway; e: flavonoid biosynthesis in L4 *vs* L0, and also in the L4 *vs* L28, L0 *vs* L13 and L13 *vs* L28 (not at the top 20 pathway); f: fructose and mannose metabolism in L0 *vs* L13; g: fatty acid degradation in L4 *vs* L0; h: fatty acid biosynthesis in L4 *vs* L28; i: phosphatidylinositol signaling system in L0 *vs* L13. The nodes are marked in red background color indicating the DEGs is up-regulated expression, and the nodes are marked in green background color indicating the DEGs is down-regulated expression. On the other side, the white nodes indicated the genes are DEGs, but there is no distinguishment of the DEGs about the up- / down-regulated expression.
