# Supplementary material for: High-throughput sequencing of small RNAs revealed the diversified cold-responsive pathways during cold stress in the wild banana (Musa itinerans)
Source: BMC Plant Biol. 2018 Nov 29;18:308. doi: 10.1186/s12870-018-1483-2 (PMC6263057; doi:10.1186/s12870-018-1483-2)
Supplement: Supplementary file 19 — Figure S7. The SOD, POD, CAT activities and H2O2 contents in the wild banana during cold stress. (PDF 1497 kb) [file 12870_2018_1483_MOESM19_ESM.pdf]

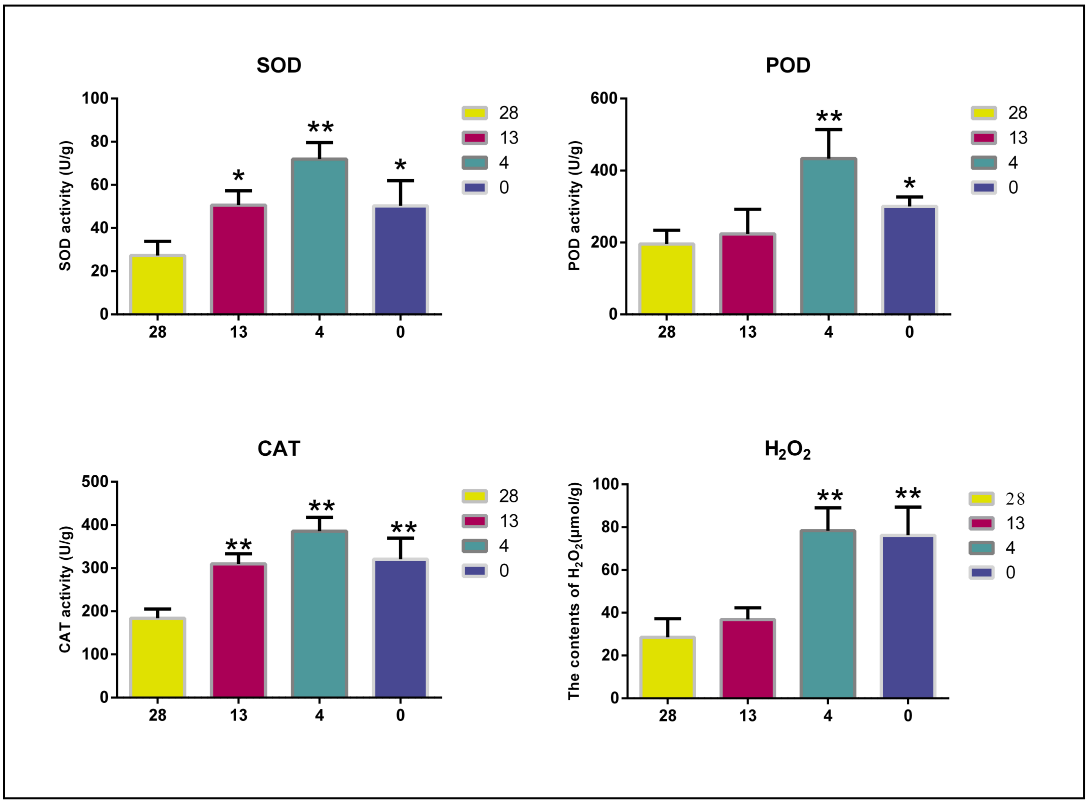

Additional file 19 Figure S7 The SOD, POD, CAT activities and H2O2 contents in the wild banana during cold stress. * showing significant differences (p≤0.05), ** showing significant differences (p≤0.01).
