# Supplementary material for: High-throughput sequencing of small RNAs revealed the diversified cold-responsive pathways during cold stress in the wild banana (Musa itinerans)
Source: BMC Plant Biol. 2018 Nov 29;18:308. doi: 10.1186/s12870-018-1483-2 (PMC6263057; doi:10.1186/s12870-018-1483-2)
Supplement: Supplementary file 20 — Figure S8. The seedlings used from treatments in the wild banana from Sanming City. (PDF 139 kb) [file 12870_2018_1483_MOESM20_ESM.pdf]

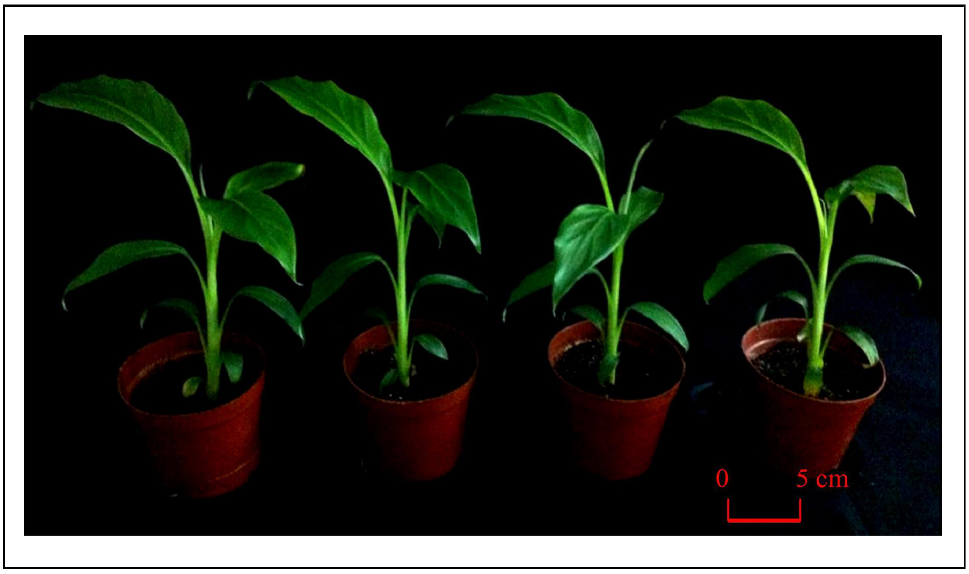

Additional file 20 Figure S8 The seedlings used from treatments in the wild banana from SanmingCity.
